# Accurate prediction of ice nucleation from room temperature water

**DOI:** 10.1073/pnas.2205347119

**Published:** 2022-07-25

**Authors:** Michael Benedict Davies, Martin Fitzner, Angelos Michaelides

**Affiliations:** ^a^Department of Physics and Astronomy, University College London, London WC1E 6BT, United Kingdom;; ^b^Yusuf Hamied Department of Chemistry, University of Cambridge, Cambridge CB2 1EW, United Kingdom;; ^c^S&T Digital Chemistry, Merck KGaA, 64293 Darmstadt, Germany

**Keywords:** ice, nucleation, deep learning

## Abstract

Crystal nucleation is one of the most fundamental processes in the physical sciences and almost always occurs heterogeneously with the aid of a nucleating substrate. No example of nucleation is more ubiquitous and impactful than the formation of ice, vital to fields as diverse as geology, biology, aeronautics, and climate science. However, despite considerable effort, we still cannot predict a priori the efficacy of a nucleating agent. Here we utilize deep learning methods to accurately predict nucleation ability from images of room temperature liquid water—generated from molecular dynamics simulations—on a broad range of substrates. The resulting model, named IcePic, can rapidly and accurately infer nucleation ability, eliminating the requirement for either notoriously expensive simulations or direct experimental measurement. In an online poll, IcePic was found to significantly outperform humans in predicting the ice nucleating efficacy of materials. By analyzing the typical errors made by humans, as well as the application of reverse interpretation methods, physical insights into the role the water contact layer plays in ice nucleation have been obtained. Moving forward, we suggest that IcePic can be used as an easy, cheap, and rapid way to discern the nucleation ability of substrates, also with potential for learning other properties related to interfacial water.

## Significance

From glaciers, to cryopreservation, to climate modeling, the ability of materials to promote ice nucleation is at the heart of a myriad of technologies and natural phenomena. Predicting the ice nucleation ability of materials thus presents great opportunity; however, it has presented an equally great challenge: despite over 75 y of research, no reliable method or guideline exists. Here we address this by developing a model that accurately predicts the ice nucleation ability of materials. Deep learning techniques enable an easy, cheap, and rapid method that requires just an image of room temperature water in contact with a substrate as input. Remarkably, the model shows that the interfacial structure of water alone is sufficient to determine nucleation.

Liquid–solid phase transitions are fundamental processes in the physical sciences. As such, the implications of their proper understanding and prediction are vast. Among innumerable candidates the formation of ice has been the subject of perhaps more studies than any other, motivated mainly by its ubiquity. In the atmosphere, ice affects cloud albedo, lifetime, and composition (1–4). This in turn makes it vital to Earth’s radiation budget and the modeling of future changes in climate (5–13). In industry, controlling ice formation presents great opportunity: cryopreservation is vital to numerous clinical applications (14), US state and local agencies spend over $2.3 billion per annum on snow and ice control (15), and deicing a single Boeing 747 airplane can cost up to ∼$50,000 (16).

It is now well established that ice formation almost always proceeds heterogeneously, whereby it is initiated by a foreign material. Experimental work has gained insight into how water structures itself on well-defined substrates (see, e.g., ref. 17) and directly measured the ice nucleation ability of different particles (see, e.g., ref. 18). Simulations have uniquely been able to provide the temporal and spatial resolution required for a molecular-level understanding (19). Together these approaches have greatly deepened our understanding; however, prediction of a material’s ability to promote or suppress ice nucleation a priori has proven to be a major challenge (19). It therefore remains necessary to determine each material’s effect on ice nucleation on a case-by-case basis. Moreover, building a detailed understanding of the ice nucleating ability of a single material often requires both experiment and simulation (20–29). The field is thus in a difficult situation: one cannot determine nucleation behavior a priori; thus, it must be established on a case-by-case basis, but even this can be extremely difficult and time consuming. This gap in understanding presents a bottleneck to innovation in industry; to relieve this a simple way to predict a material’s nucleation ability is needed.

In this work, we develop a model to accurately and rapidly predict the nucleation temperature (*T*) of a substrate without the need for direct simulation or experimental measurement of a nucleation event. The deep neural network, IcePic, uses images—generated in molecular dynamics (MD) simulations—of the first layer of water atop the substrate at room temperature as input. A broad range of substrates, representative of common inorganic ice nucleating particles, was considered. Irrespective of the substrate, values of *T* are predicted with a very low root mean squared error (RMSE) of 6.3 K and mean absolute error (MAE) of 3.7 K, and a high R^2^ coefficient of 0.91. For context, this level of predictive ability drastically exceeds that of baseline models. It also significantly exceeds the performance of humans, who were pitted against IcePic in an online poll as part of this study. Reverse interpretation methods uncover the physical information held within the images of the contact layer. These insights, combined with the archetypal errors made by humans when attempting to predict nucleation behavior from such images, lead to a guide on how the water contact layer induced by a material determines its effect on ice nucleation.

## Prediction of Ice Nucleation from Images of Water Contact Layers

**Fig. 1.**
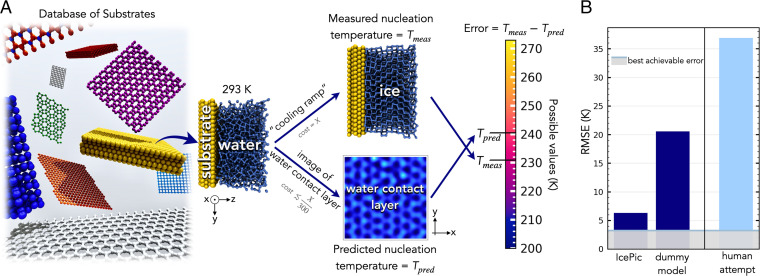
Building an image recognition model to accurately and rapidly predict the ice nucleation behavior of substrates. (*A*) Illustration of the substrate database, with an example of how ice formation is measured directly and predicted via the water contact layer and how the subsequent error in the prediction is determined. (*B*) Performance of IcePic and dummy models in predicting *T*: the best achievable error—set by the natural deviation in *T* for individual systems—and RMSE are given. An attempt by humans at this task is also reported.

Fig. 1*A* illustrates the methodology to create IcePic. The model was trained and tested across a database of 1,119 water–solid interfaces. The solid substrates considered ranged from hydroxylated surfaces with different arrangements of hydroxyl groups, to close-packed Lennard–Jones (LJ) surfaces, to graphitic and graphite oxide systems. This is by no means comprehensive but represents a broad variety of the known ice nucleating particles (INPs): the surfaces of many inorganic, organic, and biological INPs are hydroxylated; the LJ systems give a wide range of model substrates in terms of atomic roughness, symmetry, and water-substrate interaction; and there are many carbonaceous aerosols that act as INPs (18, 30). The temperature at which each substrate promotes ice nucleation –termed the nucleation temperature, *T* –was measured via cooling ramp MD simulations (*Materials and Methods*). Possible values of *T* range from 273 to 200 K. The mean SD in *T* for a substrate was ± 3 K; we set this as the best achievable (i.e., minimum) error possible for predictions. As input, IcePic takes images of the first layer of water in contact with the substrate—termed the water contact layer—at 293 K (*Materials and Methods*). The predicted value of the nucleation temperature, $T_{\text{pred}}$, is output. This can then be compared to the value measured in simulation, $T_{\text{meas}}$. Training was performed on 70% of the systems and performance tested over the remaining 30% (*Materials and Methods*).

The ability of different agents to predict *T* from such images is explored in Fig. 1*B*. It is informative to first consider the performance of a dummy model which simply returns the mean value of *T* in the training set; this informs on the triviality of the task. An RMSE of 20.74 K is achieved. The error window can be approximated by ± the RMSE. Given the window of possible *T* is ∼70 K, this represents a very poor performance. Multiple dummy models were attempted as is reported in *SI Appendix*, section S2; the best performing dummy model is reported in Fig. 1*B*.

Across the database, IcePic predicts *T* with RMSE of 6.3 K, a reduction in error of 69% when compared to the dummy model. This performance gives a small window of prediction—close to the minimum possible error of 3.0 K—that enables the accurate prediction of a substrate’s nucleation ability. A further indicator of IcePic’s accuracy is the R^2^ metric, which is the proportion of the variation in *T* explained by the model. A value of 0.91 was obtained; generally, values above 0.9 are considered to be excellent for regression models. These results indicate that an image of the water contact layer at room temperature contains enough information to accurately predict *T*. This gives a rapid computationally cheap way to infer nucleation ability, with a conservative estimate of the saving in cost being 1/300th of directly simulating with the monoatomic water (mW) model (*SI Appendix*, section S6). In the case of more complex atomistic models, simulating even a single nucleation event is notoriously difficult and expensive (19, 31), whereas it takes minimal cost to create the images to feed to IcePic.

The use of deep learning techniques means only the raw input of the images is required by IcePic. Traditionally, however, efforts into understanding and predicting a substrate’s ice nucleation ability have relied on hand-designed features such as substrate lattice match to ice (32), surface symmetry (33), and adsorption energy (a proxy for hydrophobicity) (34). A simple linear regression model utilizing such features achieved an RMSE of 12.5 K on a very similar database (approximately double the error of IcePic) (35). A more complex approach, involving screening ∼3,000 potential descriptors and a subsequent feature selection to build machine learning models, achieved a comparable RMSE to IcePic’s of ∼ 6 K but a lower R^2^ of 0.86 (35). The fact that IcePic not only matches but slightly outperforms the feature selection approach is a remarkable result as IcePic’s input is far simpler and neglects the use of any hand-designed features/descriptors produced by the community over decades of work. The most crucial benefit of utilizing deep learning here is enabling a simple input: images of water contact layers can be produced both in simulation and experiment (whereas features of ref. 35 are computational only); therefore, a potential bridge between the two disciplines has been created. We refer readers who are unfamiliar with the machine learning techniques discussed here to a pedagogical discussion on artificial intelligence techniques in *SI Appendix*, section S7.

Finally, given that IcePic demonstrated images of water contact layers can be used to predict a materials nucleation ability, a natural question arises: can humans also do this? We note that this is not meant to act as direct competition to IcePic, as it is not generally reasonable to expect humans to match performance levels of machine learning models in regression. However, given that the water contact layer is widely discussed (36, 37) and explicitly used to explain heterogeneous ice nucleation in the literature (20, 21, 23, 26, 34, 38–44)—discussion dates as far back as the 1940s with the origin of substrate lattice match (32, 45)—it is reasonable to expect some level of human expertise here. Even if it cannot match IcePic, assessing human performance can uncover gaps in our knowledge that might prove useful to address and thereby give new physical insights into ice nucleation. Therefore, both simulation and experimental researchers in the field were polled via an online survey where they were asked to label *T* for a selection of images from our database (*Materials and Methods* and *SI Appendix*, section S3). As shown in Fig. 1*B*, human performance was poor, with RMSE of 36.9 K. Such performance represents random guessing at best; at worst, it shows our current intuition is hindering performance (in other words, randomly guessing could be a better strategy). The argument for the latter is supported by the superior performance of the dummy model. However, a perhaps fairer test of human ability is to analyze the ability to rank systems in order of their overall nucleation ability (thereby removing systematic errors in the values of *T* reported). This is shown by the Spearman’s rank correlation coefficient: the mean human value was –0.11; a negative value provides further evidence that current intuition is hindering performance. In comparison, IcePic achieved +0.89. Overall, this paints a picture that inferring nucleation ability from images of the water contact layer is a highly nontrivial task and one where humans have much to learn.

## IcePic Generalizes Well to Unseen Substrates

Accurate prediction of nucleation ability from images of the water contact layer offers many benefits, not least the avoidance of extremely costly simulations and direct experimental measurement. However, confidence in the ability of IcePic to perform outside of its training data is needed. In the absence of external data, this is done by splitting the database into training and test datasets. Random stratified sampling—as reported in *Prediction of Ice Nucleation from Images of Water Contact Layers*—exposes the model to the greatest range of different substrates and values of *T* (in both training and testing), making it appropriate for producing the final model for external use. However, a more difficult assessment for the model can be executed by curating test datasets with different physical properties to the training dataset.

**Fig. 2.**
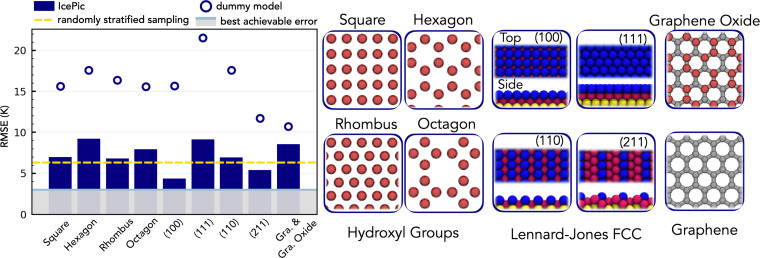
Performance of IcePic across different test datasets containing unseen structures; in each case the training dataset consists of all other systems in the database. (*Left*) Plot of IcePic’s (bars) and the dummy model’s (circles) RMSE values, along with the best achievable error (gray region) and the error achieved in random stratified sampling as reported in Fig. 1 (yellow dashed line). (*Right*) Images of a representative system from each test dataset over which the model’s performance was assessed: OH groups (red) with different tiling patterns, cuts of LJ FCC crystals (atoms colored by height), and a variety of graphene and graphene oxide like systems (carbon in gray, OH in red).

Fig. 2 summarizes the performance of IcePic when the systems were split into training and test datasets based on the substrate atomic symmetries. In each split the test dataset consists of the systems indicated (e.g., OH groups with a square tiling), and the respective IcePic model was created by training on all other systems in the database (i.e., nine versions of IcePic were created, one for each split). As expected the model performance varies as some test datasets are harder than others. For instance, the OH hexagon test dataset is particularly hard as hexagonal symmetry is known to be important to ice nucleation—as it is found on the basal face of hexagonal ice—so hiding hexagons from the model during training removes important physicochemical data. However, across all tests a consistently strong performance is achieved with RMSE values ranging between 4 and 9 K. IcePic’s maintained performance across these tougher tests indicates the model has strong generalization and should maintain performance outside of our database. For the interested reader, further regression metrics for IcePic’s performance are provided in *SI Appendix*, section S2 along with the performance of other dummy models.

## The Water Contact Layer Is Sufficient to Accurately Determine Nucleation

The fact that IcePic can determine nucleation ability is itself an interesting physical insight into nucleation. It should be noted that no information on the substrate is given to the model. Hiding substrate properties could be expected to limit the model to prediction across a single substrate type, thereby removing the variation in substrate behavior the model is lacking. However, the model is observed to predict nucleation irrespective of the particular substrate. It therefore appears that the water contact layer alone is sufficient to determine—and therefore understand—the nucleation behavior induced by substrates.

Prior to making conclusions, it is important to first address the context of the model’s training and application. Namely, it has been employed using a coarse-grained water model upon idealized model substrates. This is of great use in extracting general insights; however, it is reasonable to question the accuracy of the simulations. The model was therefore employed upon simulations from literature that can be expected to more accurately portray real INPs: AgI in contact with an aqueous electrolyte solution represented by the atomistic transferable intermolecular potential, 4 point, 2005 (TIP4P/2005) water model (46) with Na^–^ and Cl^+^ ions represented by the Madrid model (47, 48), cholesterol monohydrate (CHLM) in contact with the atomistic TIP4P/Ice water model (23, 49), and the clay kaolinite in contact with TIP4P/Ice water (28, 50). Compared to the mW water model these TIP4P atomistic simulations both have an order of magnitude increase in cost and require orders of magnitude slower cooling ramps to see crystallization, making it not computationally practical in this study to measure values of *T* for these systems. This makes it unfeasible to give a one-to-one determination between the values of *T* predicted by IcePic (presented in *SI Appendix*, Table S1) and the true value. Furthermore, using the water contact layer to recognize CHLM as a strong nucleator can be expected to be particularly challenging given that its nucleation ability is due to an “ability of flexible hydrophillic surfaces to form unconventional ice-templating structures” (23). However, the model recognizes all systems as strong INPs, orders them correctly in terms of nucleation ability, and distinguishes between the preferential nucleation behavior between the two polar faces of AgI. These statements are justified in *SI Appendix*, section S4 by comparing IcePic’s predictions to experimental and simulation studies on these systems in the literature.

The application of the model to a structurally diverse range of INPs—an inorganic crystal, an organic crystal, and a clay—has provided further evidence that the water contact layer is indeed sufficient to determine nucleation. This indicates that long-discussed metrics of the substrate such as lattice match or hydrophobicity are not necessarily required to predict nucleation. Although such metrics will of course affect the resulting water contact layer, the water contact layer alone encodes enough information to satisfactorily predict behavior.

## Deciphering the Water Contact Layer

The water contact layer is sufficient to determine nucleation, and the model can make these inferences. Therefore, removing the black box nature of the model yields the opportunity to improve our understanding of the nucleation mechanism. The scope of the possible insight is determined by the model’s learning procedure; deep learning is a type of representation learning, and here the only input has been the raw image data (pixels), from which the model learned features to determine nucleation behavior—readers who are unfamiliar with these machine learning techniques are referred to *SI Appendix*, section S7. Successfully applying reverse interpretation methods enables these features to be extracted, affording new insights. To this end, Shapley additive explanations (SHAP) were applied to IcePic (*Materials and Methods*) (51). This allowed us to assign a value to the effect on the model’s output, *T_pred_*, associated with each pixel of an image.

Materials displaying extremes of behavior in nucleation are of the greatest interest to technological application and natural phenomena; therefore, we looked to use IcePic to identify highly active and inactive water contact layers with regards to nucleation. The assignment of each pixel’s effect on *T_pred_* facilitated this: to extract images recognized by IcePic as being active for nucleation, simply take those with the largest proportion of pixels which act to increase *T_pred_* against those that decrease *T_pred_*. Similarly, taking images with the largest proportion of negative to positive pixels allows the extraction of images that IcePic recognizes as inactive to nucleation.

**Fig. 3.**
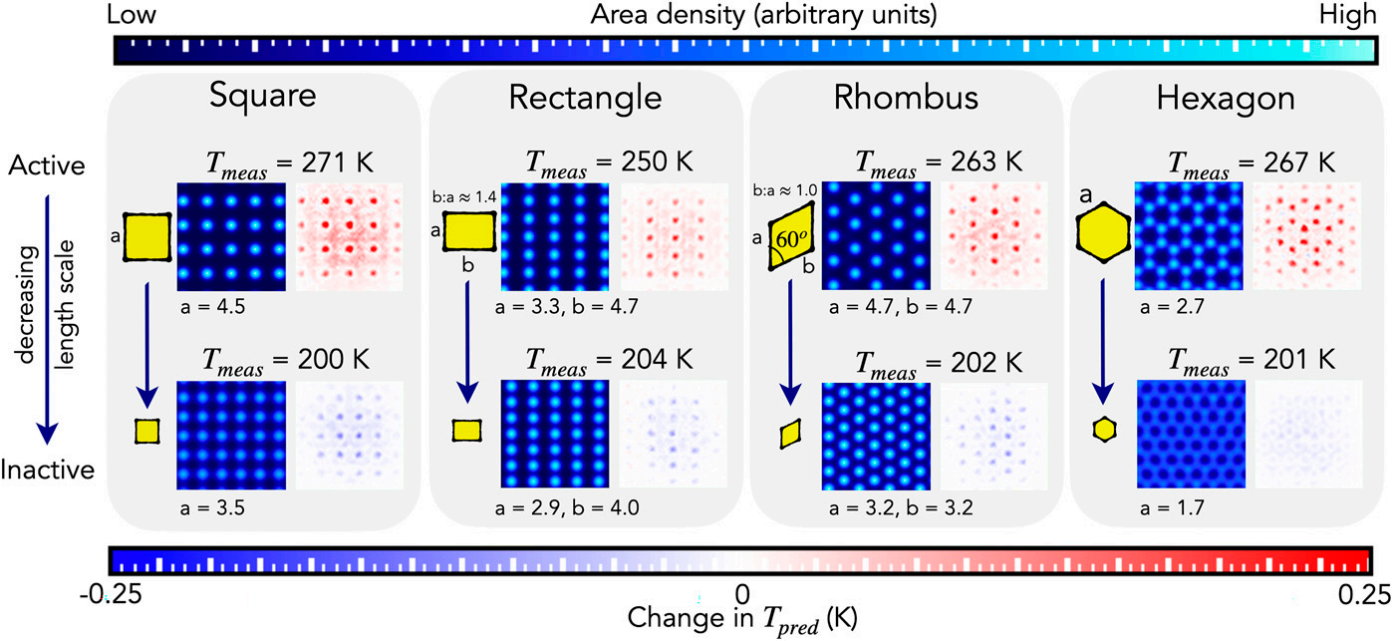
Identification of water contact layer patterns that can transition from being active (*Top*) to inactive (*Bottom*) to ice nucleation by changing their length scale. Area density images (blue color bar; *Top*) show water contact layers passed to IcePic—in each case a unit cell has been identified (square, rectangle, rhombus, or hexagon). SHAP density images (blue-white-red color bar; *Bottom*) show the same images of water contact layers but with the pixels colored by their effect on IcePic’s output: *T_pred_*.

Fig. 3 shows water contact layers composed of simple unit cells: squares, rectangles, rhombi, and hexagons. Each of these patterns can both induce and suppress nucleation. The respective ability of these patterns to promote nucleation is dependent on their length scale. They each can strongly induce nucleation, when at an optimal length scale for ice nucleation, and can each suppress nucleation, when at a length scale that is suboptimal for ice nucleation. By length scale here we refer to the magnitude of the respective unit cell vectors. This can be physically understood by the following. Nucleation is induced (high *T*) when the unit cells form water contact layers that increase the chance of a critical ice nucleus forming, which triggers the conversion of water to ice. Conversely, nucleation is suppressed (low *T*) when the unit cells form water contact layers that must undergo large rearrangement to form ice and thus impair the ability of ice nuclei to form. Each of these patterns’ dual ability to induce and suppress nucleation originates from their ability to form over a range of length scales; this allows them to transition from water contact layers that need little rearrangement to form ice (giving high *T*), to those that are far from the ice lattice and thus impair the ability of ice to form (giving low *T*).

Identifying the dual ability of these patterns highlights the importance of scale. For instance, matching a face of ice is a long-postulated way to strongly induce ice nucleation (32); however, even though the square, rhombus, and hexagonal unit cells are found on particular ice faces, they can also strongly suppress nucleation by changing their length scale. Using the patterns in Fig. 3, intelligent design and/or discovery of both potent ice promoters and inhibitors could be achieved, most obviously via substrate lattice match to the unit cell and scale that corresponds to the desired behavior.

The existence of extremes (high to low *T*) indicates a continuum of behaviors must exist—quantifying this would enable a fine tuning of nucleation behavior. To date, this would require directly obtaining such patterns (via intelligent design of substrates) and directly measuring nucleation ability (an expensive/difficult process). However, this can now be rapidly explored with IcePic by feeding artificial images to the model. This is presented in *SI Appendix*, section S5; it enables the transition from inducing to suppressing nucleation to be directly observed and quantified. Interestingly, a periodicity is predicted for all the unit cells, where nucleation ability is somewhat recovered at half length scales of the optimum. We find systems that support this prediction and postulate the recovery is due to the formation of coincidence site lattices (52, 53).

## Utilizing the Water Contact Layer: How to Infer Nucleation Behavior

So far, we have shown that the water contact layer is enough to accurately determine a substrate’s nucleation ability: proper utilization of the water contact layer could therefore give insight into INPs. However, we have also shown that humans currently lack the ability to make reliable inferences, so improving performance would be desirable. This may appear to be a difficult task; indeed, humans are generally worse at pattern recognition than deep neural networks (54–56). However, improvements can still be made. Namely, the fact that human performance was worse than dummy models indicated that our intuition is misleading us: a gap in understanding has thus been identified—one that is concerning given the widespread discussion of water contact layers in ice nucleation literature. In this section we aim to understand how water contact layers determine a materials ice nucleation ability by 1) investigating archetypal errors made by humans and 2) calling upon insights from IcePic, namely, the importance of scale.

**Fig. 4.**
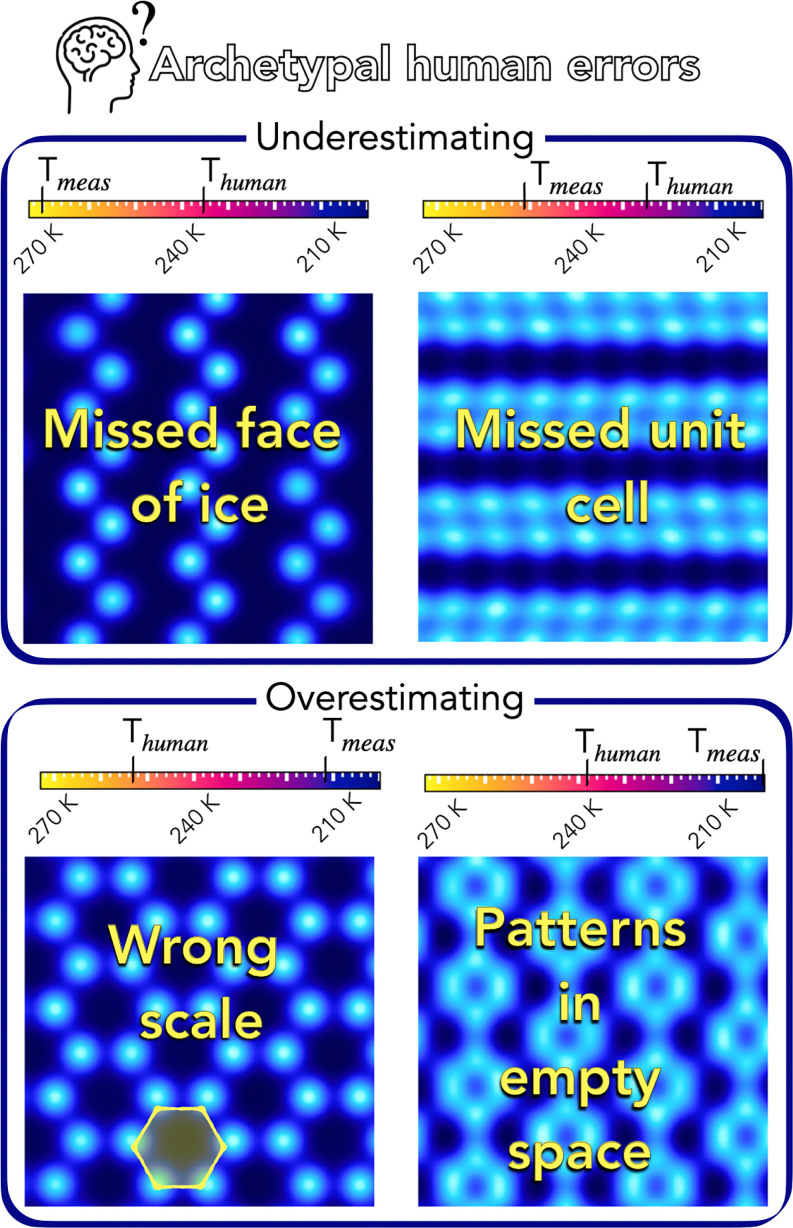
Archetypal errors made by humans when predicting nucleation temperatures. Mean values predicted for each image by quiz respondents are shown as *T_human_*.

Underestimations by humans occur primarily through failures to recognize active patterns for nucleation. We provide two examples of this in Fig. 4. The first is the easiest to address: a failure to recognize a face of ice causes large underestimations. In ref. 44, water contact layers with high similarity to any of the faces of the ice I polytypes (hexagonal, cubic, and stacking disordered) were shown to give large *T*. Failure to recognize faces (beyond, e.g., the basal face of hexagonal ice) results in huge errors; we thus emphasize the importance of considering the other ice faces. The second is harder to recognize as it is a pattern active to nucleation but does not appear to derive from a face of ice (the same is true for the rectangular unit cell of Fig. 3; we note that neither pattern matches the particular rectangular symmetry present on the prism face of ice). The solution is simply that this pattern must be noted as active to nucleation and searched for.

Conversely, two examples of overestimations by humans are provided in Fig. 4. First, a human might recognize a known unit cell for nucleation (e.g., hexagon from the basal face, drawn to correct scale in yellow in Fig. 4) and thus return a high value of *T*. However, as highlighted in *Deciphering the Water Contact Layer*, the scale of the pattern is key, and deviations can cause major changes in behavior. Second, when a known unit cell appeared, humans failed to make the distinction between it occurring in empty rather than occupied regions. It is the patterns in the water density that matter, not the patterns made by empty space.

Finally, we combine these human errors with insights from IcePic to create an approximate guide to determine how the water contact layer induced by a material determines its nucleation ability. Specifically, we suggest taking the following into consideration:

1. Look for active patterns for nucleation, both those derived and those not derived from ice.
2. If a recognizable pattern is present, determine the scale: Is the pattern matching the optimum scale? If not, is it at half length scales and thus possibly forming a coincidence site lattice? A quantitative estimate of the dependence on scale can be taken from *SI Appendix*, Fig. S5.
3. Consider deformation: does the pattern truly match an active unit cell, or is it a deformed version? A high degree of sensitivity to deformation has been observed in this study.
4. Ensure the pattern identified is occurring in the occupied regions. Patterns in empty regions are not physically there and are simply misleading.

## Discussion

In this work, we have demonstrated a deep neural network that accurately predicts the ability of substrates to promote nucleation. The model uses images of the water contact layer at room temperature as input and returns the temperature at which nucleation proceeds uninhibited, *T*. A high performance is achieved with RMSE of 6.3 K, MAE of 3.7 K, and R^2^ of 0.91. In addition, the model generalizes well to unseen substrates and appears to work well for atomistic simulations of complex solid–liquid interfaces. Predicting the nucleation ability of substrates a priori has remained an open challenge for decades. The desire originates from the innumerable impacts of ice nucleation in technology and the natural world, coupled with the extreme difficulty in measuring candidates on a case-by-case basis. Here we have obtained an easy, cheap, and rapid way to discern the nucleation ability of substrates.

The fact the model works shows that the water contact layer alone is sufficient to determine nucleation behavior, meaning that properties of the substrate are not necessarily required. This invariance to the substrate is an interesting physical insight that was shown to apply across the database as well as an inorganic crystal, an organic crystal and a clay. Furthermore, this invariance is also very beneficial to applications of the model: it reduces the required training of the model and yields strong generality. To give an example, the model is primed for screening across crystal structure databases (e.g., refs. 57–60) to find novel materials with desired nucleation abilities. Correctly ranking materials by nucleation ability is perhaps more important than error here, and confidence in IcePic’s ability to do this is given by its Spearman’s rank correlation coefficient of 0.89. Discovering novel materials is an exciting prospect for future work, with potential impacts on technological developments and our understanding of the myriad ice phenomena in nature.

A benefit of applying deep learning techniques has been enabling a simple and physically motivated input to the model that can be measured both computationally and experimentally. Traditional attempts to understand ice nucleation via computational work have relied on hand-designed microscopic quantities which cannot readily be determined in experiment. In contrast, IcePic could be deployed in experiment by feeding it images generated by scanning tunnelling and atomic force microscopy; prior work has imaged water with the necessary resolution (61–64). Predictions of nucleation behavior could be applied to—and complement—the large body of work undertaken to accurately determine how water assembles itself at interfaces (17). However, experimental images are usually taken on low-coverage or monolayer water, which can have different structures from the high-coverage contact layers used in this work (65). This may affect IcePic’s performance, but if this proves true, it can readily be addressed by training a similar model on monolayer images of water.

Using reverse interpretation methods enabled insights into how to infer nucleation behavior from the water contact layer, something that our polling revealed humans lacked the ability to do. Specifically, unit cells with a dual ability to promote and suppress nucleation were identified. This highlights the importance of scale. Feeding artificial images enabled the transition to be quantified, which in turn enables a fine tuning of nucleation behaviors: decide on the desired behavior, locate the unit cells and scales associated, and design or search for substrates that would give this. Furthermore, the feeding of artificial images serves as an example of how such a model can be utilized to rapidly answer queries of interest. Combining these insights with the archetypal errors made by humans allowed a guide to be created on how the water contact layer induced by a material determines its nucleation ability.

With regard to discovery or intelligent design of INPs, this work formulates this as a two part problem: 1) determine the water contact layer given by the material and 2) use the water contact layer to predict nucleation behavior. The model could thus form part of future composite models—by playing the role of deciding whether a design is good or not for nucleation—that could, for example, be used to design ice-inhibiting surfaces for aeronautics and ice-promoting surfaces for geoengineering. Composite models have been a widespread success in artificial intelligence, with famous recent examples being the AlphaZero (66) and AlphaFold (67) models, to name just a few.

Finally, with the hope that future work will utilize and extend this study, we have released the model, and the code to generate the model and inputs at https://doi.org/10.17863/CAM.81078. The code is also available on GitHub at https://github.com/mbdavies13/IcePic. We invite interested researchers to try the model on their simulation and/or experimental data. Providing further systems to the model in training, and thus further physics, would inevitably help overall performance and generality. Furthermore, extending to other systems of interest and/or properties is an exciting avenue for future work. The methodology presented here can readily be employed on the countless systems of interest in physical and materials science. The approach removes the requirement for creating hand-designed descriptors entirely, thus enabling the rapid development of models for prediction of properties. Initial efforts will focus on those where a close relationship to the water structure is expected such as dynamical properties (diffusion, viscosity, and friction). Connection between properties could then also be studied; for instance, if transfer learning or multioutput regression improves performance compared to isolated models, then this would provide strong evidence for causative links [e.g., between nucleation and dynamics (68)]. Extending to areas other than water/ice where a better understanding of nucleation is also highly sought after, such as pharmaceutical drug design, colloids, and fine chemicals, would be of great interest. One could also use the method to train a model to predict the heterogeneous nucleation rate (*J*), an observable that has the benefit of being measurable in both experiment and simulation. However, determination of *J* over a large corpus of systems (a requirement to generate the model’s training data) could be challenging due to the computational cost/complexity of extracting *J*, which typically requires enhanced sampling techniques (19, 31).

## Materials and Methods

### MD.

All MD simulations from which *T* has been determined have been reported in earlier studies (35, 44). Heterogeneous nucleation was modeled as indicated in Fig. 1*A*, whereby water molecules were placed in contact with a slab of substrate, periodic in (*x*, *y*). The substrate database consists of 1,119 different substrate–water systems: the LJ systems from ref. 34; graphitic and graphite oxide surfaces modeled in a manner similar to those in refs. 43 and 40, respectively; the OH group patterns from ref. 33; and 219 LJ and OH group systems generated in ref. 44. As detailed in ref. 44, simulations were performed with the large-scale atomic/molecular massively parallel simulator code (69), used the coarse-grained mW water model (70), sampled the constant number of particles, constant volume, and constant temperature (NVT) canonical ensemble, and determined *T* over five cooling ramps per system. Simulation boxes are ∼ 45 to 60 Å in (*x*, *y*), with 4,000 to 6,000 water molecules, giving layers of 35 to 60 Å thickness.

### Images of the Water Contact Layer.

To generate images, NVT simulations at 293 K were performed for 4 ns and sampled every 0.5 ps, after an initial equilibration of 0.5 ns. The water contact layer was defined via the water number density profile in z: specifically the region enclosing the largest peak in the density. An example of this with further details is provided in *SI Appendix*, section S1. Images are generated by placing a rotationally symmetric normalized two-dimensional Gaussian at the location of each atom, upon a fine grid with spacing 0.01 nm. The image is normalized by the total number of trajectory frames. In atomistic simulations, positions of the oxygen atoms of water molecules were taken, and hydrogen atoms were neglected; this ensured consistency between the coarse-grained and atomistic simulations. This can also be consistent with experiment; for instance, atomic force microscopy techniques have been employed to generate high-resolution images of water that predominantly measure oxygen atoms, with only modest contributions from the hydrogen atoms (61). If solutes were present, as in the AgI simulation, they were included in the images. Final images were taken to have dimension (200, 200) which physically represents 2 nm in each dimension, at a resolution of 0.01 nm.

### Machine Learning.

The primary version of IcePic—reported in Fig. 1—was trained on 70% of the systems and performance tested over the remaining 30%; the split was performed with random stratified sampling such that the distribution of *T* is conserved, and performance was averaged over six of these splits.

Convolutional neural networks (CNNs) were used in this study and were built using the Python libraries Keras, which is freely available at (https://keras.io), and Tensorflow (71). As input, the networks take images with dimensions of (50) with a single channel, resized from the original (200, 200) images generated in simulation, resulting in a physical resolution of 0.04 nm per pixel. Basic building blocks consist of sequential convolutional layers, followed by batch normalization, and then a maximum pooling layer; this approximately follows the architecture of visual geometry group (VGG) blocks (72). Further details on the network architectures are detailed in *SI Appendix*, sections S7 and S8 and Table S2. CNNs were fitted using the Adam optimizer (73) with the mean squared error as the loss function. Deep neural networks can have high variance due to the stochastic nature of their training; thus, final predictions were taken by combining multiple models into committees (or ensembles) and taking the mean of their predictions. To ensure rotational and translational invariance, data augmentation techniques were employed on the training database, whereby rotated and translated versions of images were given to the model; this is also a well-known technique to ensure greater generality in the features learned by CNNs and thus improve accuracy in image recognition.

SHAP is a game theory approach, which utilizes a power set of a model’s features to decompose the impact of each feature on the model’s output (51). This was applied to IcePic to decompose the relationship between the model inputs (image pixels) and the resulting output (*T_pred_*). This enables each pixel of an image to be assigned a quantitative value on its effect on *T_pred_*.

### Human Poll.

Human performance was recorded via an online quiz which asked people to label *T* from a selection of images from our database. In total, 59 responses were received. Detailed results are provided in *SI Appendix*, section S3. The overall average of the scores is reported in Fig. 1. A PDF file of the quiz web page along with the correct answers is provided.

The poll was approved by the University of Cambridge following the guidelines established by the University Research Ethics Committee. All respondents gave informed consent before participating.

## Supplementary Material

Supplementary File

Supplementary File

## Data Availability

All data and code that support the findings of this study are openly available at the University of Cambridge Data Repository (Apollo; https://doi.org/10.17863/CAM.81078) (74). The code to generate the models and inputs has also been deposited in GitHub (https://github.com/mbdavies13/IcePic) (75). All data needed to evaluate the conclusions in the paper are present in the paper and/or the *SI Appendix*.
