# Supplementary material for: Accurate prediction of ice nucleation from room temperature water
Source: Proc Natl Acad Sci U S A. 2022 Jul 25;119(31):e2205347119. doi: 10.1073/pnas.2205347119 (PMC9351478; doi:10.1073/pnas.2205347119)
Supplement: Supplementary File [file pnas.2205347119.sapp.pdf]

PNAS
www.pnas.org

Supplementary Information for

Supporting Information: Accurate Prediction of Ice Nucleation From Room Temperature Water.

Michael Benedict Davies, Martin Fitzner, Angelos Michaelides

Angelos Michaelides.
E-mail: am452@cam.ac.uk

This PDF file includes:

Supplementary text
Figs. S1 to S11
Tables S1 to S2
Legend for Dataset S1
SI References

Other supplementary materials for this manuscript include the following:

Dataset S1

Supporting Information Text

1. Definition of the water contact layer

The definition used in this work for the water contact layer is illustrated in Fig. S1.

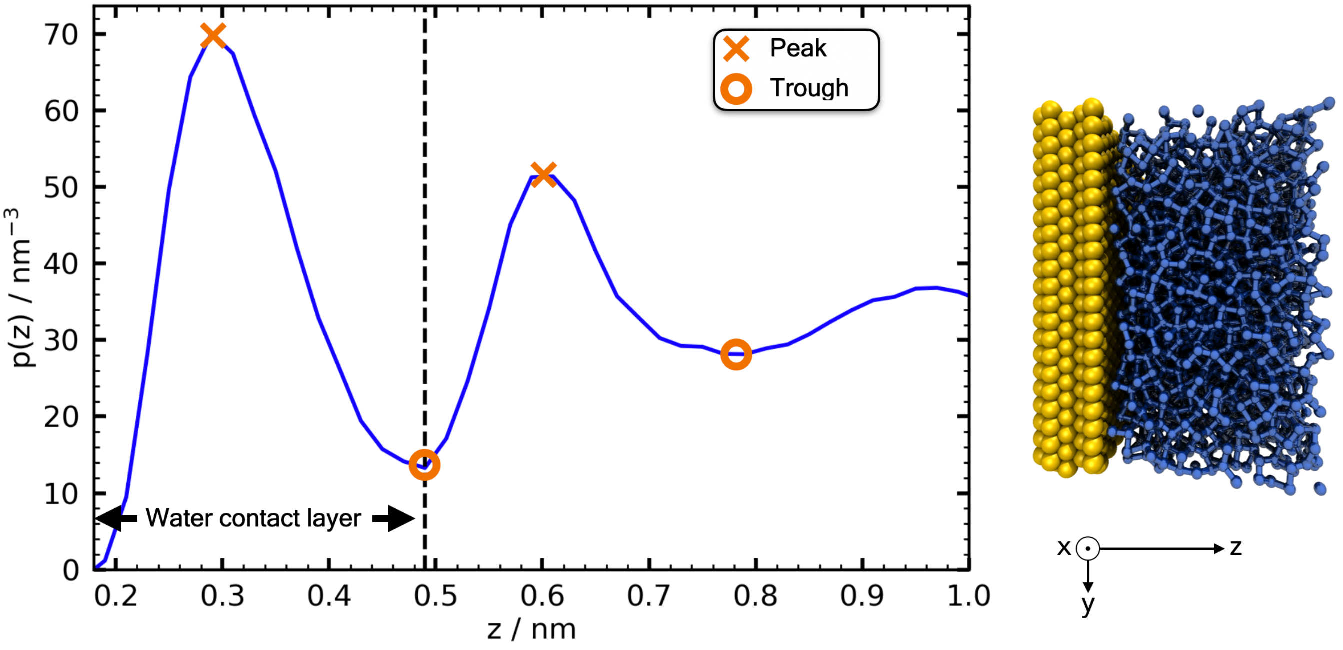

Fig. S1. Example of the definition of the water contact layer. The water contact layer is defined as the region up to the first trough after the largest peak in the number density of water molecules atop the substrate, p(z). To allow for doublets, if the peak after the largest peak is within 5 % of it or the trough after the largest peak is higher than 40 nm^{-3}, the subsequent trough in p(z) is taken as the upper limit in z. Atom positions in the xy-plane are taken from this region to build up the water contact layer images.

2. Further analysis of regression performance

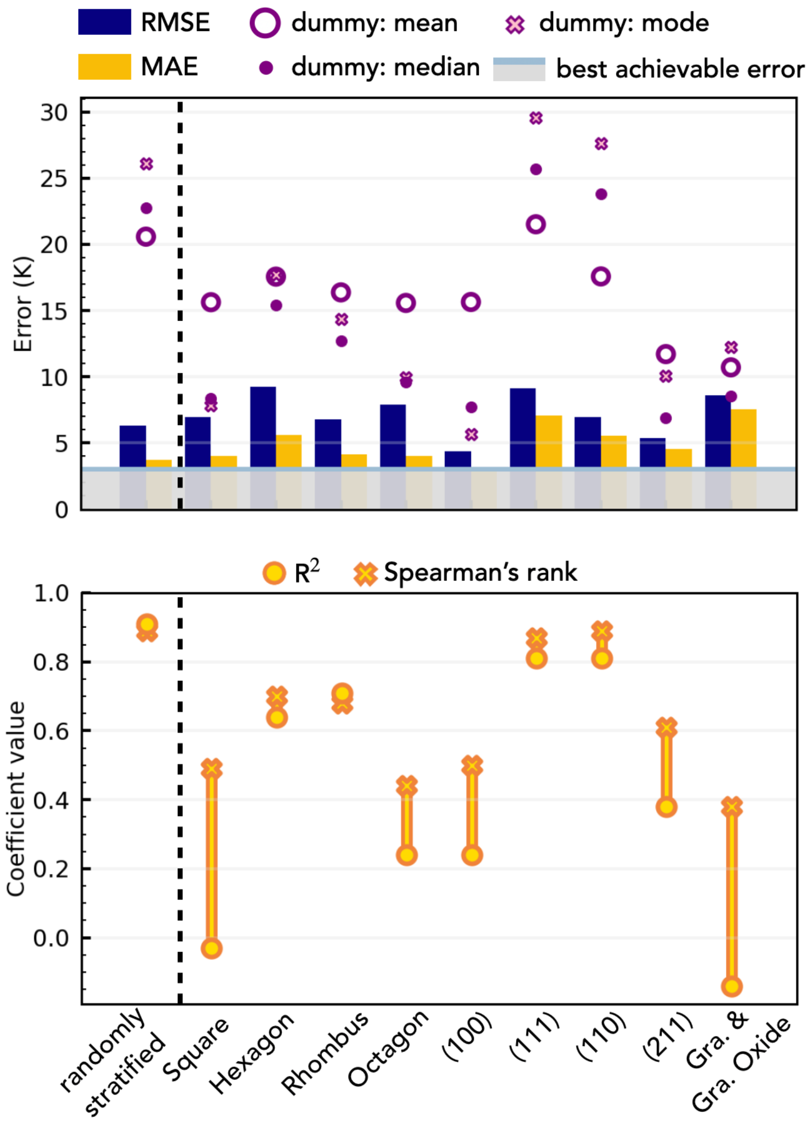

Fig. S2. Detailed analysis of regression performance of IcePic across all the test databases presented in the main text. (top) Error metrics: bar plots show the RMSE and MAE of IcePic, along with the RMSE of dummy models returning the mean (open circles), median (filled circles), and mode (crosses) of the respective training data. (bottom) Regression coefficients: R^2 and Spearman's rank coefficients for IcePic; values for dummy models are not shown as they are effectively meaningless for models that return a single output value.

Fig. S2 provides further breakdown of the regression performance of IcePic and the dummy models for the different test databases presented in the main text. The test database created via random stratification is that used to create the version of IcePic for external use – the result is averaged over 6 splits of the training/test database. For the sake of brevity one dummy model was reported in the main text: "dummy mean" as this provided the best performance on the stratified databases.

The regression coefficients, R^2 and Spearman's rank, have large variation across the different splits in Fig. S2; and for many splits the coefficients are poor despite a satisfactory RMSE and MAE. This apparent discrepancy between the error metrics and the regression coefficients comes from the change in size of the window of possible T. For a constant value of RMSE and MAE, decreasing the size of this window will cause an inevitable drop in the values of the coefficients. Thus the RMSE and MAE values are a more consistently informative metric here for model performance. The R^2 and Spearman's rank are informative for the stratified database where the window of T values is largest.

3. Human performance at predicting T

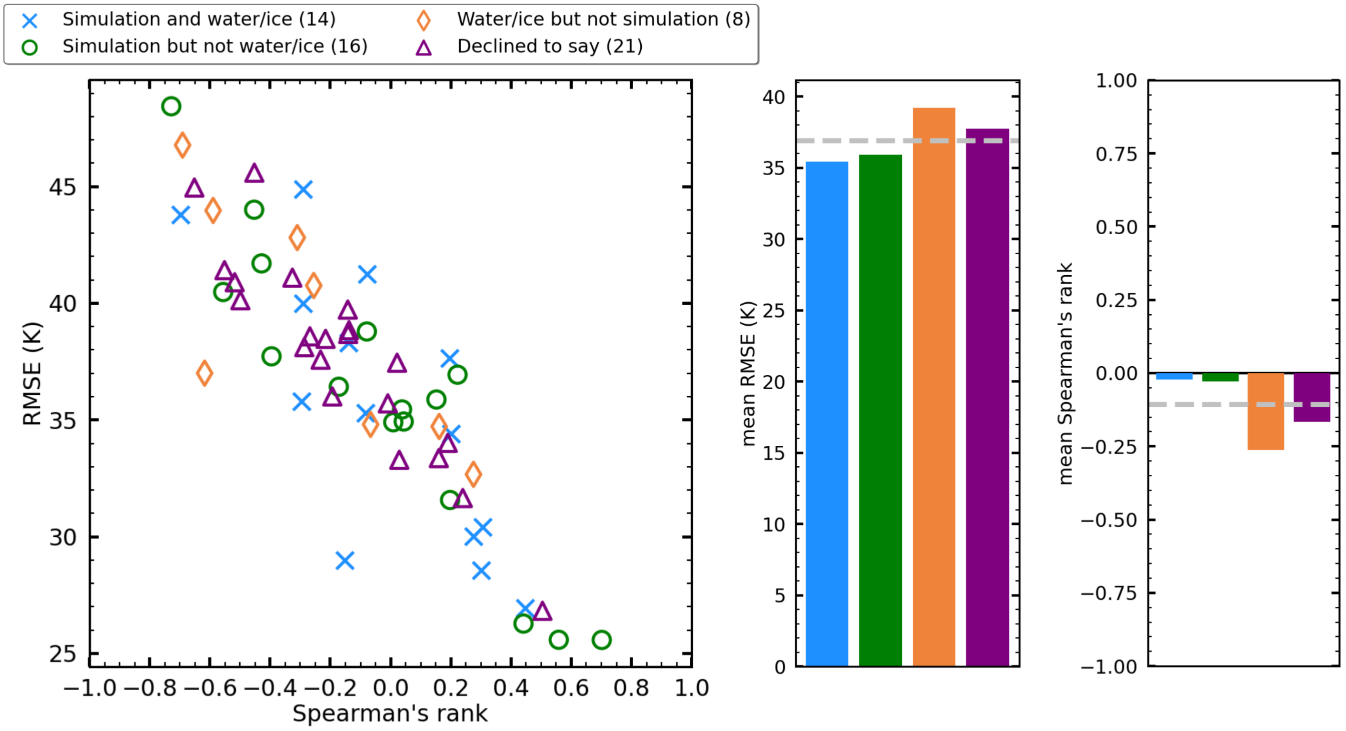

Fig. S3. Breakdown of human performance in predicting T. In total 59 people responded and these are grouped into four categories based on their research experience: (i) both in molecular simulation and studying water/ice/nucleation; (ii) in molecular simulation but not in studying water/ice/nucleation; (iii) in water/ice/nucleation but not in molecular simulation; and (iv) those who declined to provide this information. Note, no respondents identified themselves as not having experience in both simulation and water/ice/nucleation. Scatter plot shows each respondents RMSE and Spearman's rank correlation coefficient. Bar plots show the mean values of each group obtained for RMSE and Spearman's rank – grey dashed lines indicate the mean values across all respondents.

Human performance was recorded over 15 images taken from the database. Images were chosen to approximately recreate the distribution of T found over the entire database, and taken from a variety of substrate types – scale bars were provided as aid. Respondents were asked to label T for each image, one after the other. The question order was randomised for each respondent to reduce the systematic bias that might arise from fatigue building as a respondent progressed through the quiz. Multiple submissions by a single person were not allowed, and this was enforced via HTTP cookies. Respondents had to provide answers for all of the images, and as integers in the possible range of values. They were also asked their background via two questions: (i) "have you ever done molecular simulations?"; (ii) "have you ever performed scientific work on water/ice/nucleation?". This allowed us to group respondents into categories. Respondents were gathered via direct invitation, advertising via department newsletters and bulletin boards, and social media. Upon submission respondents were provided with their RMSE and MAE, however correct answers for the questions were not published until the quiz was closed.

Fig. S3 shows the performance of humans in predicting T. One would expect those with a background both in simulation and water/ice/nucleation to perform best; however, their performance appears very similar to those with experience in simulation but not in water/ice/nucleation. The Kolmogorov-Smirnov test is a mathematical non-parametric test to compare the similarity of two continuous one-dimensional probability distributions. This was applied to compare the distribution of RMSE values between the two aforementioned groups. A value of 0.1875 was obtained for the Kolmogorov-Sminrov statistic, with an associated p-value of 0.8965. This indicates a high probability that the two samples originate from an identical population. That is to say, there is no appreciable difference in behaviour between the two groups and it appears their performance is the same. The fact that researchers with experience in simulation have the same performance regardless of whether or not they have experience in water/ice/nucleation further indicates that current intuition into this problem is not helping performance.

4. Deploying IcePic on atomistic simulations

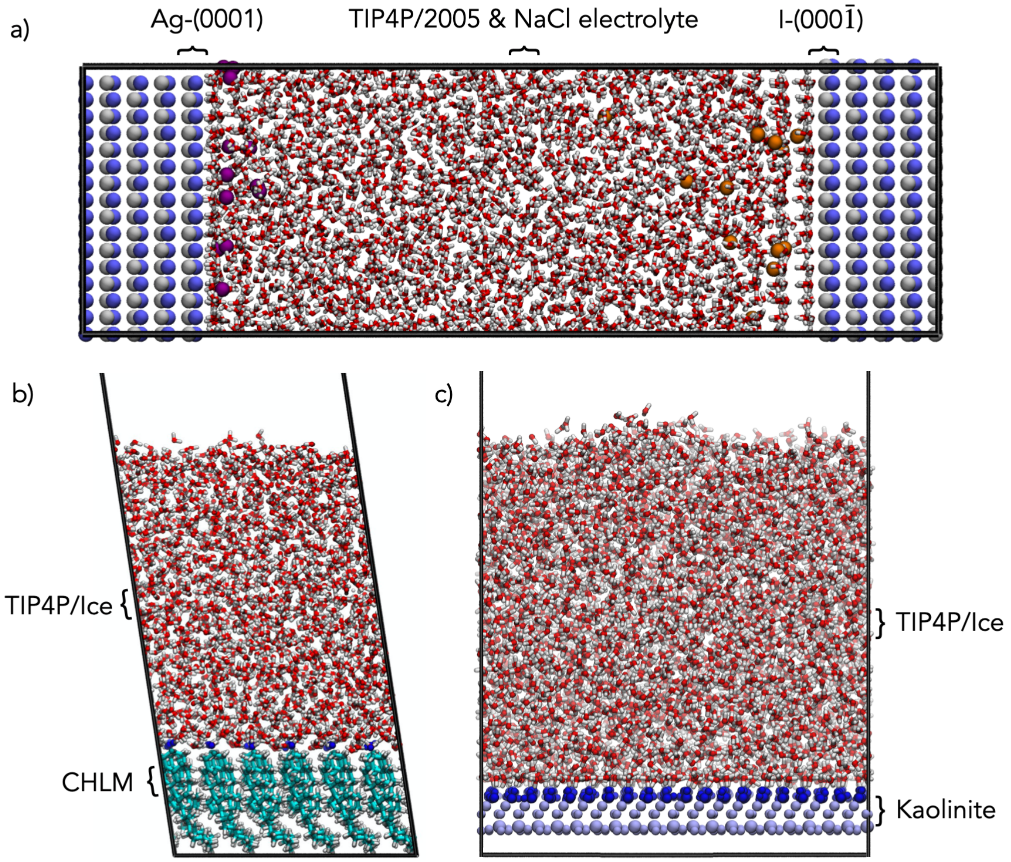

Fig. S4. Snapshots of atomistic simulations upon which IcePic was deployed. Water molecules shown as ball and stick models with oxygens in red and hydrogens in white. a) AgI crystal in contact with aqueous electrolyte solution with water represented by the TIP4P/2005 model and NaCl represented by the Madrid model. AgI is composed of alternate layers of Ag^+ and I^- ions. The positively charged Ag-(0001) and negatively charged I-(000$\bar{1}$) surfaces are exposed to the electrolyte. Ag^+ (silver), I^- (blue), Na^+ (purple), Cl^- (orange). b) Slab of cholesterol monohydrate (CHLM) with its hydroxylated (001) face in contact with a water film of TIP4P/Ice water molecules. Carbon atoms in green and CHLM hydroxyl groups purple. c) Slab of kaolinite (with all atoms coloured light blue for simplicity) with its hydroxylated (001) face (coloured purple) in contact with a water film of TIP4P/Ice water molecules.

Table S1. Predictions by IcePic on atomisitc simulations of INPs. Errors are given by one standard deviation of predictions between the six committee models deployed.

	AgI			
	Ag-(0001)	I-(000$\bar{1}$)	CHLM	Kaolinite
T_{pred} (K)	253 ± 2	242 ± 3	243 ± 3	241 ± 3

IcePic was deployed on atomistic simulations taken from the literature (see *Methods*) as depicted in Fig. S4. Table S1 shows the predictions of IcePic for the respective systems. We note that the simulations of CHLM and kaolinite employed here did not have the substrates frozen in position, as is the case for all other systems in this work. The flexible substrates cause the converged images taken over 4 ns (as done elsewhere in this work) to be unrepresentative of the instantaneous water contact layer present at any time; even a stable pattern will undergo translational movement due to the substrate flexibility which will "smear" the image as it is built from individual snapshots averaged over time. Therefore, images were taken over shorter timescales for CHLM and kaolinite. Specifically 5 ps was used; this timescale was chosen as it results an image that shows the hexagonal pattern that is known to form on kaolinite (1) whilst minimising the effects of the "smearing" caused by the flexible substrate.

As stated in the main manuscript, measuring values of T for these atomistic simulations is not feasible in this study, and there is not a one-to-one mapping between T and other metrics in the literature such as the nucleation rate. Thus comparison between IcePic's T and a true value is not done here. However, predictions of T can be used to make the following conclusions (as discussed in the main manuscript).

First, IcePic identifies all three systems as being active to nucleation, with values of T that are significantly above that of 200 K which would obtained for inactive surfaces. The model has therefore correctly predicted that these materials will reduce the barrier to ice formation and thereby promote heterogeneous ice nucleation.

Second, IcePic ranks the systems correctly in terms of their ability to promote ice nucleation. Experimentally, the respective abilities of these INP systems can be determined via immersion freezing experiments where droplets of water are exposed to the INP, the temperature is then ramped down and the fraction of droplets that are frozen is measured as a function of temperature. This has been done for AgI (2), CHLM (3) and kaolinite (4). The higher the temperature at which all droplets freeze (i.e. frozen fraction = 1) the greater the ability of the INP to promote ice nucleation. Values of $\sim$ 258 K, $\sim$ 252 K, and $\sim$ 239 K respectively were observed for AgI, CHLM and kaolinite (note, in each case we have reported the value obtained for the largest particles measured as our simulations use periodic boundary conditions to mimic a infinitely large slab of the material). IcePic qualitatively agrees with this ranking, placing AgI first (note the more potent face, Ag-(0001), is the relevant value for such experiments: both faces will be exposed to water and the most active will be the active site for nucleation), CHLM second and kaolinite third. But one must note that IcePic gives T for CHLM and kaolinite systems within each others error bar, thus it can only suggest CHLM is slightly better than kaolinite and can not make a strong conclusion on this point. However the blame here does not lie with IcePic but with a standing disagreement between experiment and simulation. The nucleation rates reported from the forward flux sampling simulations undertaken on these setups for CHLM and kaolinite are $10^{27\pm3}$ and $10^{26\pm2} s^{-1} m^{-3}$ respectively (3, 5). This similarly pits CHLM as slightly better than kaolinite but within the error bar. Resolving this discrepancy between experiment and simulation for these two INPs is a matter external to this study.

Third, IcePic correctly identifies that ice formation proceeds preferentially on the Ag-(0001) face over that of the I-(000$\bar{1}$) face as observed for this setup (6) and in other simulations of AgI in the literature (7, 8).

5. Investigating scale dependence of unit cells: Recovery of nucleation behaviour at half length scales.

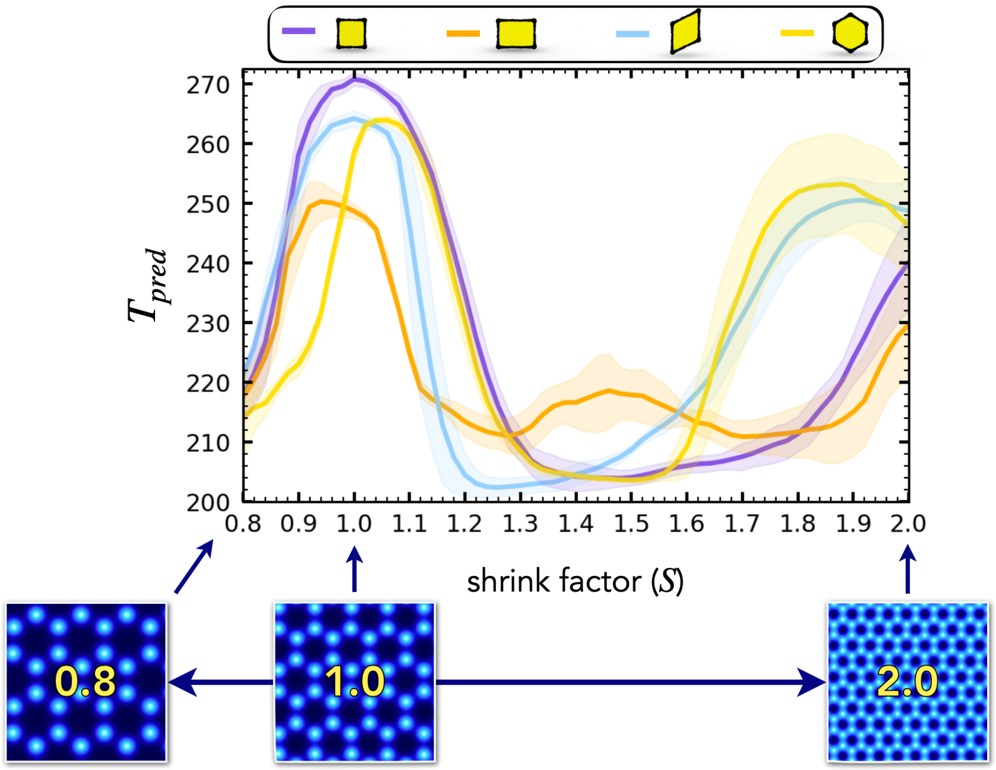

Fig. S5. Predictions of IcePic for scaled versions of water contact layers that are made by unit cells (square, rectangle, rhombus, hexagon) as identified in Fig. 3 of the main article. Plot of T_{pred} for scaled versions of patterns – the shrink factor (S) indicates reduction of the length scale. For each water contact layer pattern the original image (S of 1) upon which the scaling is subsequently performed is from the active pattern of Fig. 3; an example of this for the hexagonal pattern is shown. Shaded regions around lines indicate error bars taken as one standard deviation from the predictions of the committee models employed.

Fig. S5 shows the predictions of IcePic for scaled version of the patterns identified in Fig. 3 of the main manuscript. Predictions of T are gathered by via feeding artificial images to the model. The original scale ($S = 1$) images are taken from the "active" systems shown in Fig. 3 of the manuscript. The length scale of the patterns are then adjusted via S. This is analogous to zooming in and out of the image; however, here the width of the gaussians that represent the atoms is also scaled with S such that they have the same physical dimensions as the original image.

Care must be taken when deploying machine learning models to make predictions that can't be directly validated. However, the use of committee models here allows one to understand how uncertain the model is in the prediction it is making: the larger the deviation in predictions from each member of the committee then the larger the uncertainty. Regions of low uncertainty in Fig. S5 are likely where the model has seen similar images already: most obviously this occurs at $S = 1.0$, where the original image is fed to the model prior to any scaling. Care should be taken when making inferences from regions where the model has large uncertainty in its predictions. We move now to discuss the results obtained.

It is interesting to note that the pattern's peaks at $S \approx 1$ are symmetric in the x-axis. This indicates the depression in nucleation ability is approximately the same whether reducing or increasing the distance between water molecules in the contact layer away from the optimal distance. In general the peaks are also quite narrow which indicates that care must be taken to accurately determine the scale of an observed water pattern prior to concluding on the nucleation behaviour it may induce: relatively small changes can have a large effect on behaviour.

It is also interesting to note the recovery made by all patterns at half length scales ($S \approx 2$). The origin of such a nucleation temperature rise could be explained by the formation of "coincidence site lattices" (9, 10) – where the shrunken unit cell shares many lattice points with a full scale pattern and thus recovers its epitaxial ability. However, as stated care is needed when using machine learning models without direct validation. We therefore looked into our database to see if water contact layers at such length scales could be found; reasonably well matching systems were found for the rhombic and the hexagonal patterns.

Fig. S6 explores this for the rhombic unit cell. Fig. S6a shows the rhombic pattern at half-scale forms a coincidence site lattice both with itself and the full hexagonal pattern. We postulate this is the origin of the recovered nucleation temperature. This is supported by a system with a water contact layer very close to the half-scale rhombic pattern shown in Fig. S6b. This half-scale rhombic pattern does indeed give high T, with a value of 251 K being recorded. Furthermore, the hexagonal pattern matches the basal face of ice. Thus, if a coincidence site lattice with the hexagonal pattern from the basal face of ice is the cause of the systems rise in T then one would expect to see this face of ice form; Fig. S6c shows this is indeed the case.

Similarly, Fig. S7 explores this for the hexagonal unit cell. Fig. S7a shows the hexagonal pattern at half-scale forms a coincidence site lattice with the full hexagonal pattern. A system with such a half-scale hexagonal water contact layer is shown in Fig. S7b, and this indeed gives a high value of 253 K for T. Again, the basal face of ice forms on the system: in agreement with the formation of a coincidence lattice for the full scale hexagonal pattern.

To conclude, the example systems shown in Fig. S6 and S7 provided validation for IcePic's prediction of regained nucleation ability at half length scales. Although exact matching systems were not found for the sqaure and rectangle systems in our database it is reasonable to expect a similar physical mechanism there. Future work could look to validate this by designing systems to give such water contact layers.

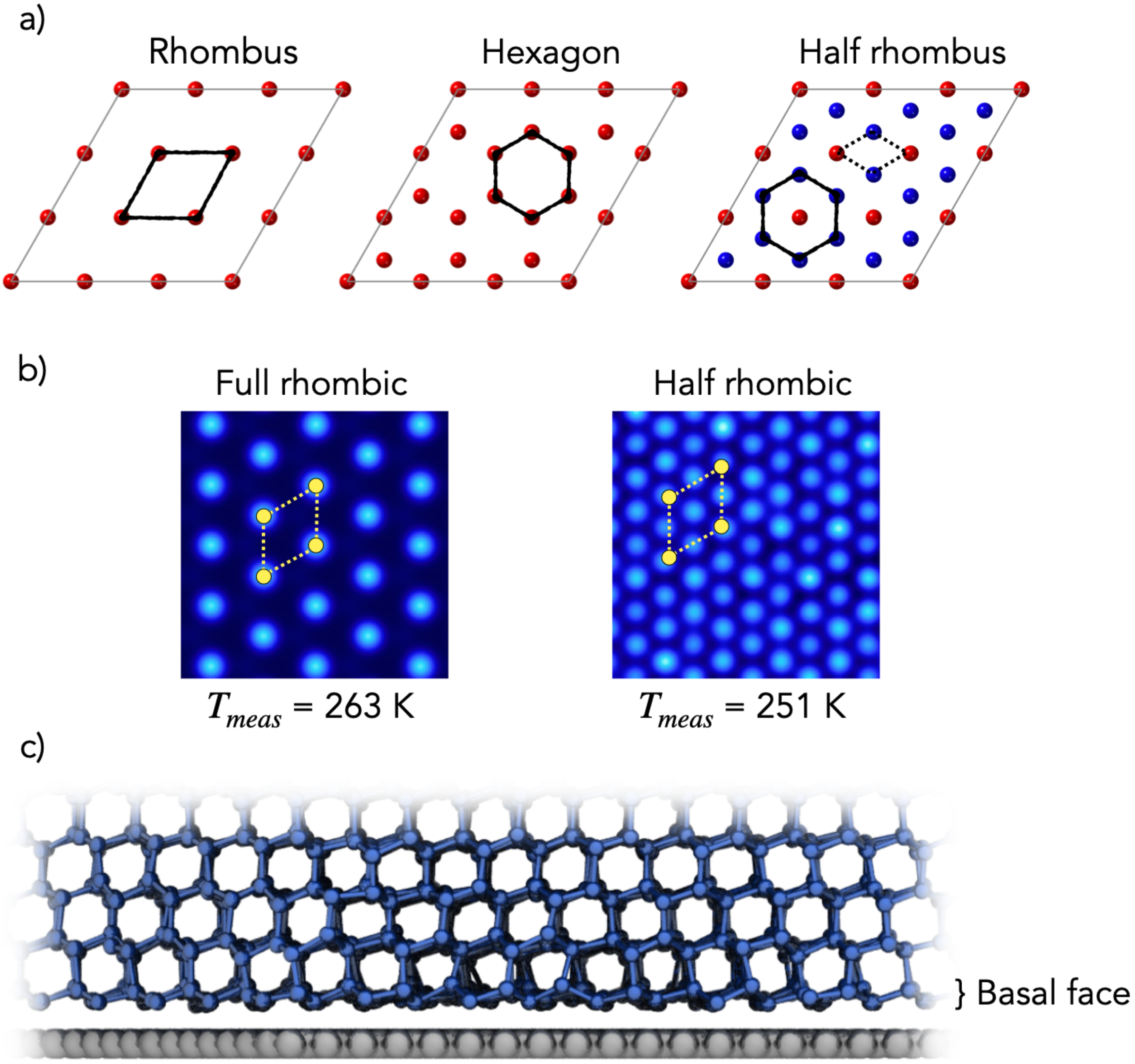

Fig. S6. Recovery of nucleation performance at half scales of rhombic unit cell. a) Schematic of original scale rhombic and hexagonal patterns, and half scale rhombic pattern. At half scale the rhombic pattern forms a coincidence lattice with the full scale hexagon unit cell. b) Example water contact layer with full and half rhombus scale. Measured nucleation temperatures are given. c) Snapshot of ice formation on the half rhombus system. The basal face of ice forms on the example system: in agreement with the formation of a coincidence lattice for the hexagonal pattern.

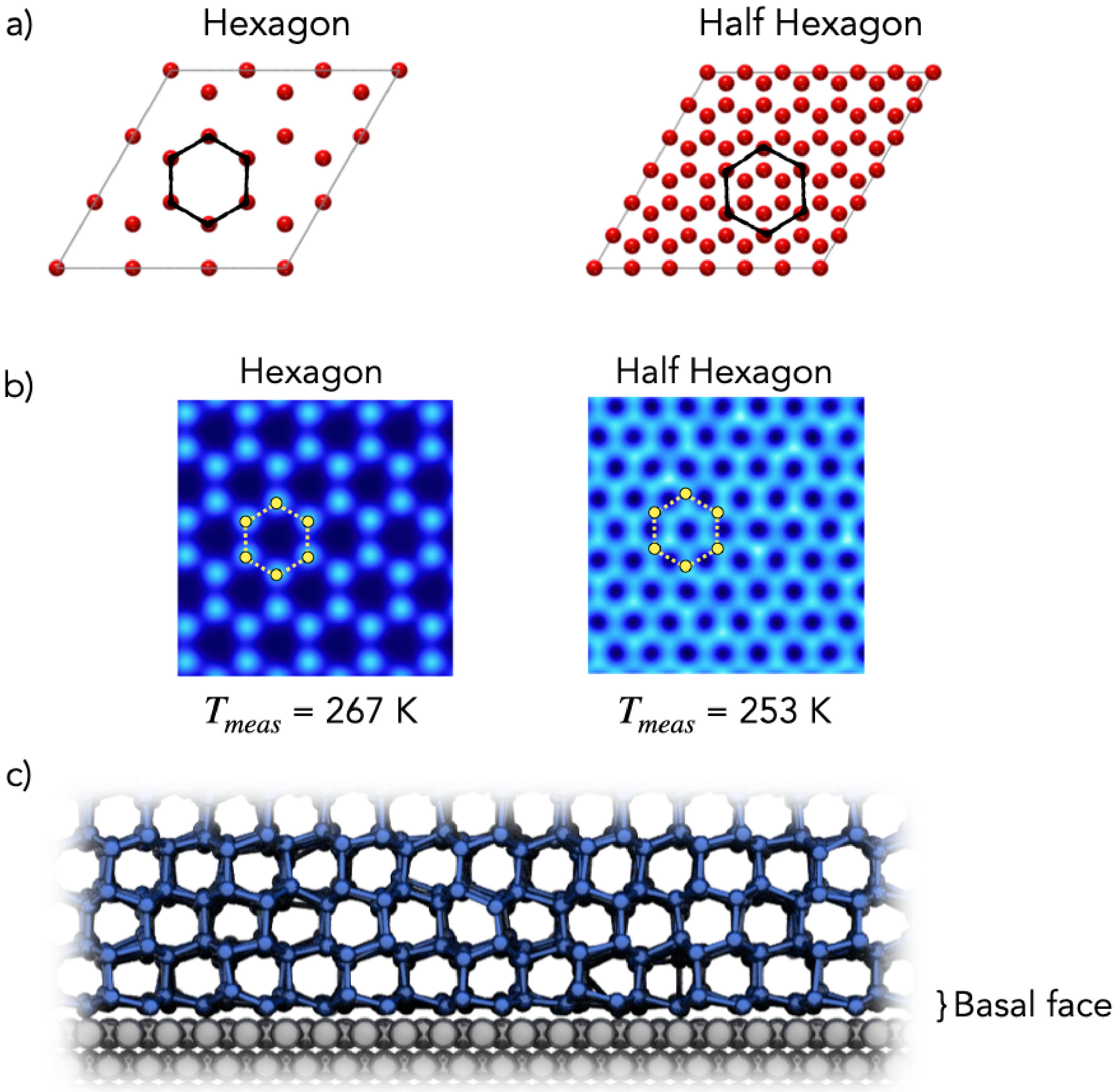

Fig. S7. Recovery of nucleation performance at half scales of hexagonal unit cell. a) Schematic of original and half scale hexagonal patterns. At half scale the hexagonal pattern forms a coincidence lattice with the full scale hexagon unit cell. b) Example water contact layer with full and half hexagonal scale. c) Snapshot of ice formation on the half hexagonal system. The basal face of ice forms on the example system: in agreement with the formation of a coincidence lattice for the hexagonal pattern.

6. Cost of image generation versus nucleation simulation

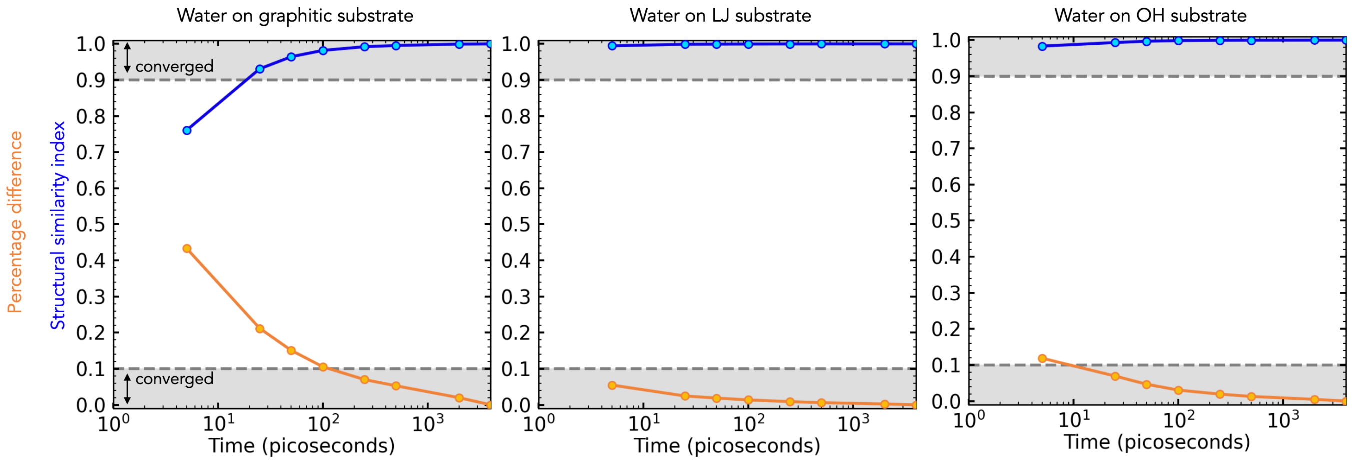

Fig. S8. Convergence of images of water contact layers with time, across a selection of systems from the database (substrates are graphitic, LJ atoms, and OH groups) Two metrics are plotted, the percentage difference between the images (orange) and the structure similarity index (blue). The latter is a well-known and reliable measure for the similarity of two images (11). The former is provided due to its conceptual simplicity. Metrics are taken against the image obtained from 4,000 ps. Grey regions indicate the threshold for satisfactory convergence (greater than 90 % similar or less than 10 % different).

The majority of the computational cost of generating images versus that of directly simulating nucleation lies in the respective simulations required – any additional costs such as creating the image or extracting nucleation information is negligible in comparison.

In this work production runs of 4 ns were used to create images of the water contact layer. However, this was done for the sake of caution to ensure converged images were acquired, and is probably far longer than required. Therefore, the simulation time required to generate converged images of the water contact layer is explored in Fig. S8. For the three system types the longest time observed for both metrics to have crossed the threshold for convergence is 100 ps. A conservative estimate of time needed for convergence would thus be 250 ps (= 0.25 ns). In comparison cooling ramps should be run at maximum rate of 1 K/ns to ensure local thermodynamic equilibrium; cooling ramps thus require 75 ns of simulation to run across the full range of temperatures. The cost of generating the images is therefore $\approx 1/300$th the cost of simulating nucleation via a cooling ramp with the mW water model.

The results above are for simulation of the mW water model. As stated prior observing nucleation in atomistic simulations remains immensely expensive and challenging, often requiring enhanced sampling methods. In comparison images of the water contact layer can be generated with just 100's of ps of relatively simple NVT simulations.

7. A pedagogical section on the artificial intelligence techniques employed in IcePic.

For the benefit of readers who have had little exposure to machine learning, here we provide a brief informative discussion on the artificial intelligence techniques employed in this study. This is by no means comprehensive, and is simply meant to provide more context to the techniques used. We refer the interested reader to the excellent textbooks of refs. (12, 13), from which much of this discussion is based, to learn more.

Machine learning is fundamentally the use of algorithms and statistical methods to make predictions and draw inferences from data. This is achieved by creating computer systems ("machines") that are able to learn and adapt their behaviour from data. The details of "how" and "what" the machine learns defines much of the approach, success and potential to draw inferences.

In this study, the machine is "IcePic". And "what" IcePic learns is the mapping from the input (the water contact layer) to the output (nucleation temperature, T), as illustrated in Fig. S9. "How" IcePic learns this mapping is by providing it with example pairs of inputs and outputs: this is referred to as "supervised learning". The database of these (input, output) pairs forms IcePic's "training data". As illustrated in Fig. 1 the predictions of IcePic (T_{pred}) can be compared to the true values extracted from simulations (T_{meas}), to give a measure of the error in IcePic's mapping. Learning a more accurate mapping then effectively becomes an optimisation task, where the total error of IcePic's predictions over its training data are minimised by repeatedly updating the mapping.

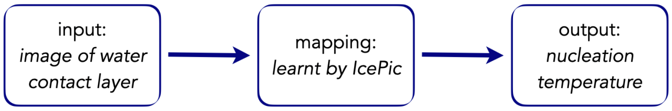

Fig. S9. A simple illustration of what IcePic learns.

In any type of predictive modelling, the representation of the data used to make predictions is fundamental to the viability of building a model with satisfactory performance. The set of representations that one uses to make predictions are often referred to as the "features". With regards to feature generation, there are two broad disciplines that machine learning can be divided into: "traditional" machine learning and "representation learning" – these are illustrated in Fig. S10.

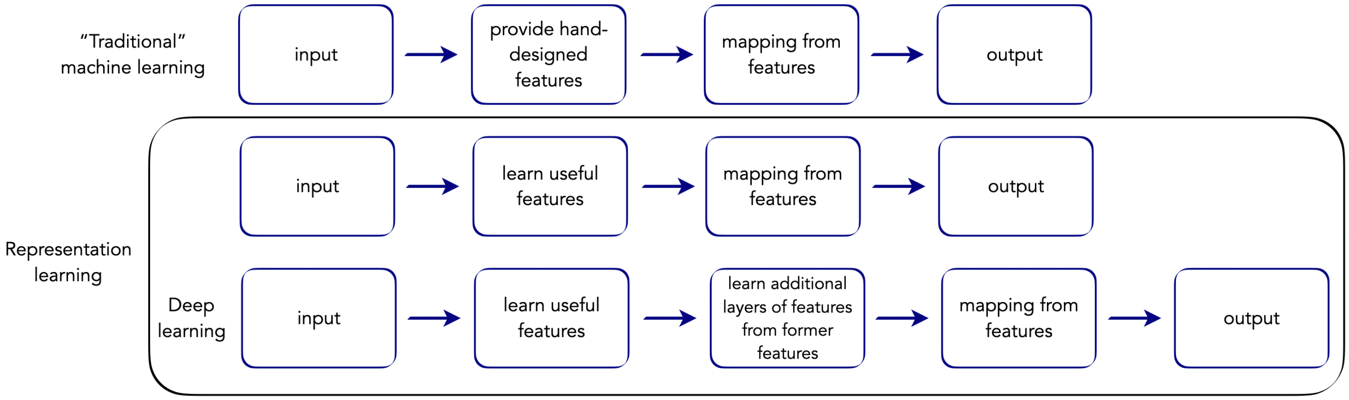

Fig. S10. An illustration of some machine learning disciplines.

In traditional machine learning, one provides hand-designed features to the model (often via intuition, domain knowledge and extensive investigation). Examples of such features attempted in the ice nucleation literature include the substrate lattice match to ice (14), surface symmetry (15), and adsorption energy (16). Success is reliant on generating a competent feature set which gives a representation of the data that enables a mapping from input to output, and this can be very challenging. Heterogeneous ice nucleation is a prime example of this, where *a-priori* prediction of a substrate's nucleation ability has remained an open challenge for decades despite extensive work.

Representation learning offers a solution to this problem. Here, machine learning is used to learn not just the mapping from representation to output, but also the representation itself. In other words: the model learns its own feature set, as well as how to map the feature set's representation of the data to the output. "Deep learning" is a type of representation learning which is performed in a hierarchical manner: multiple layers of representations are learnt, each expressed in terms of the former. This enables the machine to combine simple features into evermore complex overall concepts – instead of having to immediately extract high-level abstract features.

IcePic is a deep learning neural network; more specifically a convolutional neural network (CNN). Its input is the raw pixel data of an image of the water contact layer. The CNN then employs deep learning to break the complex mapping of input to output, into a nested series of simpler mappings. This is illustrated in Fig. S11; the convolutional filters learnt by the model form the feature set. A quintessential example of this – that is conceptually easy to understand – is using a CNN for face

detection in images, where for example the first hidden layer might detect edges, the second uses this to detect corners, the third uses this to detect facial features (e.g. nose, eyes, mouth) which is then used to determine if a face is present or not in the image. In the current application, building an understanding of what the filters represent is more challenging; CNN filters are inherently quite abstract, and when applied to the abstract images of water contact layers it is hard to understand what they represent. However, other reverse interpretation techniques can be applied to draw insights on how the model makes inferences. In this study, the application of SHAP values proved fruitful in extracting insights about the nucleation process: specifically unit cell symmetries and length scales.

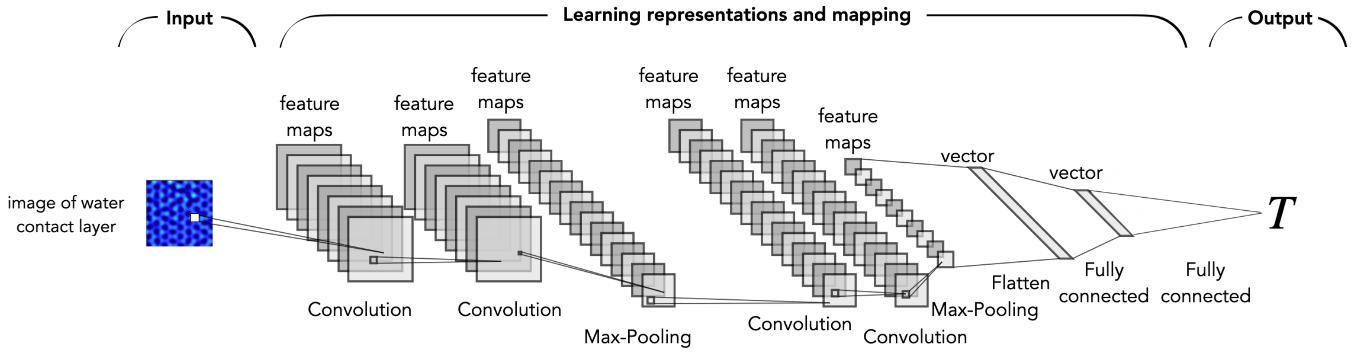

Fig. S11. Schematic of a possible CNN structure for IcePic. An example water contact layer image is fed to the CNN. In the first layer, multiple convolutional filters are passed over the image to each give a filtered version of the image; this forms a feature map. A single filter being passed over the image is illustrated by a small square that connects it to the first layer. The size and number of filters is chosen by hand. Subsequent layers are then generated by filtering these feature maps with more convolutional filters – generating further feature maps. Max-pooling calculates the maximum values of patches of a feature map; it is periodically used to extract more general features and give some invariance to local translations. The filters are learnt by the model during the supervised learning training procedure; together they form the features learnt by the model, which yield nested representations that enable the successful mapping of the input to the output. The final feature map is flattened into a vector such that it can be input to the fully connected layers – these act like a traditional artificial neural network and combine the features generated by the convolutional layers to give the final predicted value of T. This schematic is of a structure that has been simplified for the sake of clarity, and is not a complete diagram of an IcePic structure; batch normalisation and dropout have not been illustrated, see *Materials and Methods* for how this was incorporated. Image made with NN-SVG Software (17).

For the sake of brevity we have not explained all the technical details of a CNN model, and we refer the interested reader to ref. (12) and the wealth of literature on the topic. The aim here has been to introduce the general concepts such that the implications for the application at hand might be better understood. The use of deep learning has removed the requirement for generating hand-designed features, and yields a methodology that can easily be applied to other quantities of interest (e.g. dynamics and friction).

8. Architecture of CNNs employed

Table S2. Architectures of convolutional blocks of CNNs

no. of VGG blocks	filter size	no. of filters in sequential blocks
3	3x3	32, 64, 128
3	5x5	32, 64, 128
4	3x3	32, 64, 128, 256
4	5x5	32, 64, 128, 256
5	3x3	32, 64, 128, 256, 512

Table S2 details the different architectures of the convolutional parts of the CNNs utilised in this study. Filters are passed with a stride of 1 and padding to maintain dimension sizes between inputs and outputs, and the rectified linear activation function is used. Upon applying all VGG blocks, the data is flattened and input to a fully connected layer, where dropout regularization with rate of 0.5 is applied prior to the final layer where a linear activation function is used.

As stated in the manuscript, committees of models were used to make predictions. The different architectures shown in Table S2 were used to increase the differences between models in the committees, thereby giving more uncorrelated predictions between them.

SI Dataset S1 (SI_quiz.pdf)
The nucleation quiz, along with answers, is provided as a pdf file.

References

1. GC Sosso, GA Tribello, A Zen, P Pedevilla, A Michaelides, Ice formation on kaolinite: Insights from molecular dynamics simulations. *The J. Chem. Phys.* **145**, 211927 (2016).
2. C Marcolli, B Nagare, A Welti, U Lohmann, Ice nucleation efficiency of AgI: review and new insights. *Atmospheric Chem. Phys.* **16**, 8915–8937 (2016).
3. GC Sosso, et al., Unravelling the origins of ice nucleation on organic crystals. *Chem. Sci.* **9**, 8077–8088 (2018).
4. BJ Murray, SL Broadley, TW Wilson, JD Atkinson, RH Wills, Heterogeneous freezing of water droplets containing kaolinite particles. *Atmos. Chem. Phys.* **11**, 4191–4207 (2011).
5. GC Sosso, T Li, D Donadio, GA Tribello, A Michaelides, Microscopic Mechanism and Kinetics of Ice Formation at Complex Interfaces: Zooming in on Kaolinite. *J. Phys. Chem. Lett.* **7**, 2350–2355 (2016).
6. T Sayer, SJ Cox, Stabilization of AgI's polar surfaces by the aqueous environment, and its implications for ice formation. *Phys. Chem. Chem. Phys.* **21**, 14546–14555 (2019).
7. G Fraux, JPK Doye, Note: Heterogeneous ice nucleation on silver-iodide-like surfaces. *The J. Chem. Phys.* **141**, 216101 (2014).
8. SA Zielke, AK Bertram, GN Patey, A Molecular Mechanism of Ice Nucleation on Model AgI Surfaces. *The J. Phys. Chem. B* **119**, 9049–9055 (2015).
9. W Bollmann, *Crystal defects and crystalline interfaces.* (Springer Science & Business Media), (2012).
10. W Bollmann, On the geometry of grain and phase boundaries. *The Philos. Mag. A J. Theor. Exp. Appl. Phys.* **16**, 363–381 (1967).
11. Z Wang, AC Bovik, HR Sheikh, EP Simoncelli, Image quality assessment: from error visibility to structural similarity. *IEEE Transactions on Image Process.* **13**, 600–612 (2004).
12. IJ Goodfellow, Y Bengio, A Courville, *Deep Learning.* (MIT Press, Cambridge, MA, USA), (2016).
13. G James, D Witten, T Hastie, R Tibshirani, *An introduction to statistical learning: with applications in R.* (Springer, New York, NY, USA), (2013).
14. B Vonnegut, The Nucleation of Ice Formation by Silver Iodide. *J. Appl. Phys.* **18**, 593–595 (1947).
15. P Pedevilla, M Fitzner, A Michaelides, What makes a good descriptor for heterogeneous ice nucleation on OH-patterned surfaces. *Phys. Rev. B* **96**, 115441 (2017).
16. M Fitzner, GC Sosso, SJ Cox, A Michaelides, The Many Faces of Heterogeneous Ice Nucleation: Interplay between Surface Morphology and Hydrophobicity. *J. Am. Chem. Soc.* **137**, 13658–13669 (2015).
17. A LeNail, NN-SVG: Publication-Ready Neural Network Architecture Schematics. *J. Open Source Softw.* **4**, 747 (2019).
