# Supplementary material for: Accurate prediction of ice nucleation from room temperature water
Source: Proc Natl Acad Sci U S A. 2022 Jul 25;119(31):e2205347119. doi: 10.1073/pnas.2205347119 (PMC9351478; doi:10.1073/pnas.2205347119)
Supplement: Supplementary File [file pnas.2205347119.sd01.pdf]

The ICE Group

Ice nucleation showdown: Humans vs AI

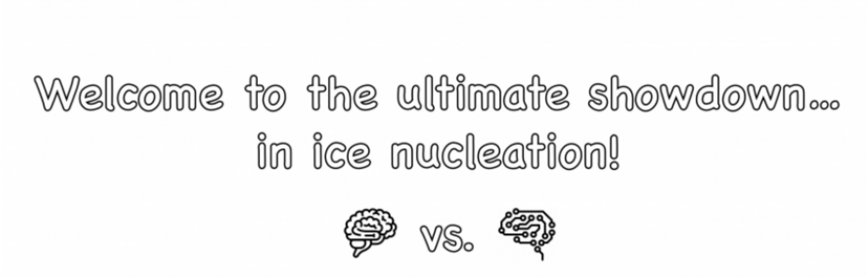

Take on our ice nucleation AI machine!

Time needed to complete: ~ 5 mins

Introducing the showdown you never knew you needed... In the red corner, our all-new AI ice nucleating machine - in the blue corner, the challenger, is you!

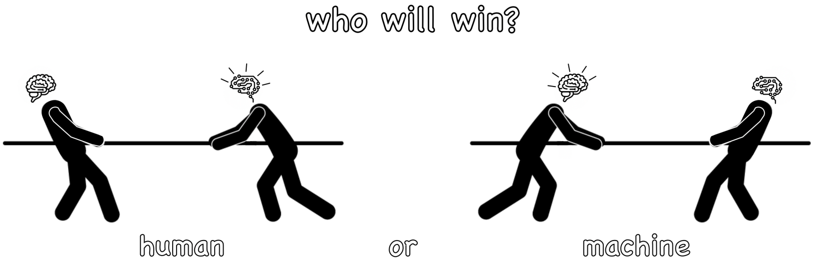

Your nemesis: Our AI machine has been built using state of the art convolutional neural networks. It's trained and thinks it's ready to take you on in a head-to-head battle!

Essential details: We've undertaken simulations of water on top of a broad range of solid substrates. By running "cooling" ramps, where the temperature is lowered from 275 K to 200 K at 1 K/ns, we have obtained the temperature at which ice formation proceeds uninhibited for each system -- the "*nucleation temperature*".

What you and the AI are given: images of the first layer of liquid water in contact with each substrate, at room temperature.

What you and the AI are not given: any information on the substrates.

Your challenge: use the image of the water contact layer to predict the *nucleation temperature* for each system.

Possible answers: the worst systems have a nucleation temperature = 200 K, the best systems have a nucleation temperature = 273 K. This gives the range of possible answers.

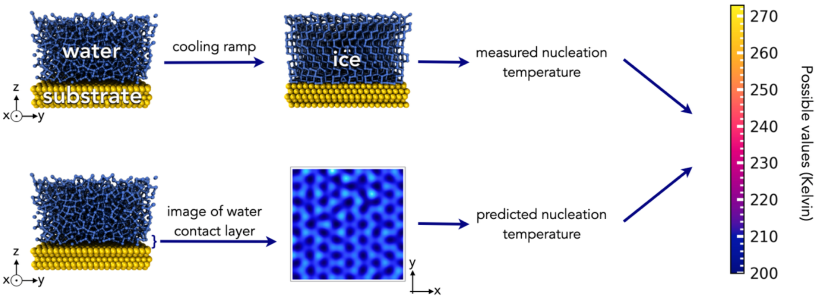

Important information: no personal data will be collected during this quiz other than the option to provide your email to receive your score. Anonymised consolidated responses may be included in future publications.

Click to start the quiz

Details of the quiz:

The motivation: over 50+ years of research has been undertaken to understand how ice nucleation is

The motivation: over [illegible] years of research has been undertaken to understand how ice nucleation is promoted (or inhibited) by foreign materials ("substrates"). The community has built up quite a bit of expertise over the decades... however, AI techniques are replacing human experts across innumerable disciplines. So, we've built a new AI machine to challenge our understanding of this fundamental phenomena.

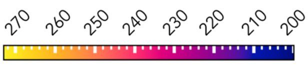

Possible nucleation temperature lies in the range 200 to 273 K: you'll need to provide answers as integers within this range.

There will be 15 images in total, each representing 2 nm x 2 nm (scale bars are provided).

If you're confident you understand the premise, then you can start now.

Click to start the quiz

If you'd like more background then please keep reading...

Background:

In nature, the nucleation of ice almost always proceeds in the presence of a foreign substrate. The substrates structure the water in contact with them: exactly how they structure the water can have a large effect on the nucleation process, and thus the value of the nucleation temperature (T).

Nucleation is promoted (high T) when the water contact layers are structured in a way that optimizes the conversion of water to ice.

Conversely, nucleation is suppressed (low T) when the water contact layers must undergo large rearrangement to form ice, and thus impair the ability of ice nuclei to form.

Tip: water contact layers which are similar to a face of ice have been shown to give high T (ref. 1). However, exceptions to this "lattice match" rule have been shown (e.g. ref 2).

Our simulations:

Simulations were performed as detailed in (1). Specifically, the coarse-grained mW water model was employed, and the solid substrates considered range from hydroxylated surfaces with different patterns of hydroxyl groups, to close-packed Lennard-Jones (LJ) surfaces, to graphitic and graphite oxide systems. However, as already mentioned, you will receive no information on which substrate the water contact layer has come from in the quiz. Your challenge is to use the water contact layer alone to predict T.

The water contact layer:

These were generated via constant temperature simulations, at 293 K. The water contact layer is defined via the density profile in z: specifically the region up to the trough after the largest peak in the density signal as shown below.

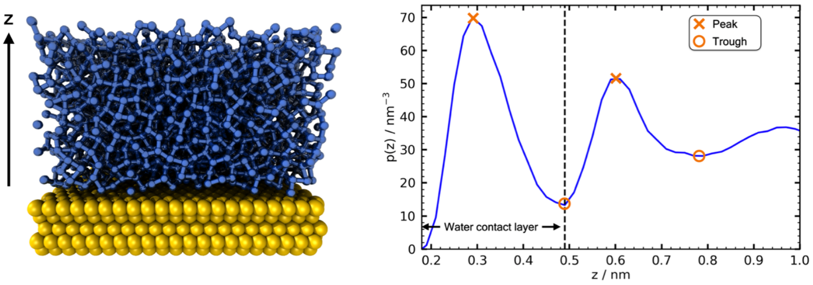

Schematic of how the water contact layer is defined from the density profile in z, p(z), of the water atop the substrate.

Click to start the quiz

If you have any concerns or questions, please don't hesitate to contact us: md899@cam.ac.uk

(1) M. B. Davies, M. Fitzner, and A. Michaelides, "Routes to cubic ice through heterogeneous nucleation", Proceedings of the National Academy of Sciences, vol. 118, p. e2025245118, 2021

(2) M. Fitzner, P. Pedevilla, and A. Michaelides, "Predicting heterogeneous ice nucleation with a data driven approach", Nature Communications, vol. 11, no. 1, p. 4777, 2020

Related Publications

Routes to cubic ice through heterogeneous nucleation
MB Davies, M Fitzner, A Michaelides – Proc Natl Acad Sci U S A **(2021)** *118,* e2025245118
(DOI: 10.1073/PNAS.2025245118)

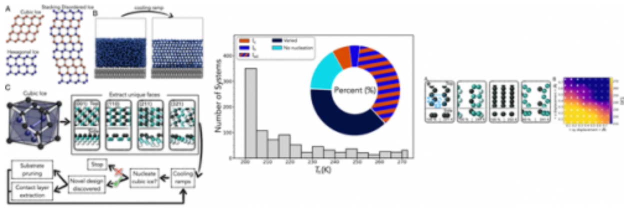

Welcome to the ultimate showdown... in ice nucleation!

VS.

Time needed to complete: ~ 5 mins

Your challenge:
Use each image of the first layer of water atop a substrate to predict the *nucleation temperature* for the respective system. **Reminder: the substrate is not shown.**

There will be 15 images in total, each representing 2 nm x 2 nm (scale bars are provided).

Thank you for taking the time to complete the quiz.

Important information: no personal data will be collected during this quiz other than the option to provide your email address if you wish to recieve your results. Anonymised consolidated responses may be included in future publications.

Start

Powered by
Opinio Survey Software

1. **Please let us know your background.**

- ☐ Have you ever done molecular simulations?
- ☐ Have you ever performed scientific work on water / ice / nucleation?

2. **If you'd like to see your results then please provide your email address in the box below: we will email them to you.**

   If not then leave the box below blank and your response will stay anonymous.

   p.s. if you wish to remain anonymous then we still strongly encourage you to take part! All responses will contribute to the human vs AI battle, and will help build new insights in this vital area of research.

2/17

Back Next

Powered by
Opinio Survey Software

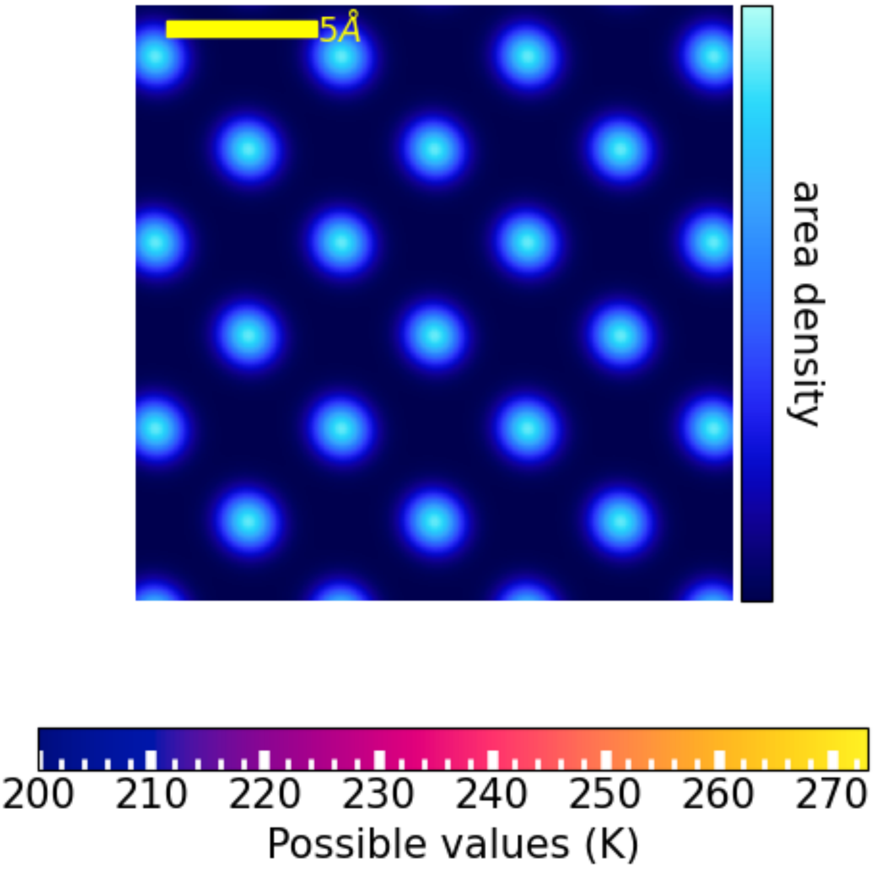

5Å
area density
200
210
220
230
240
250
260
270
Possible values (K)

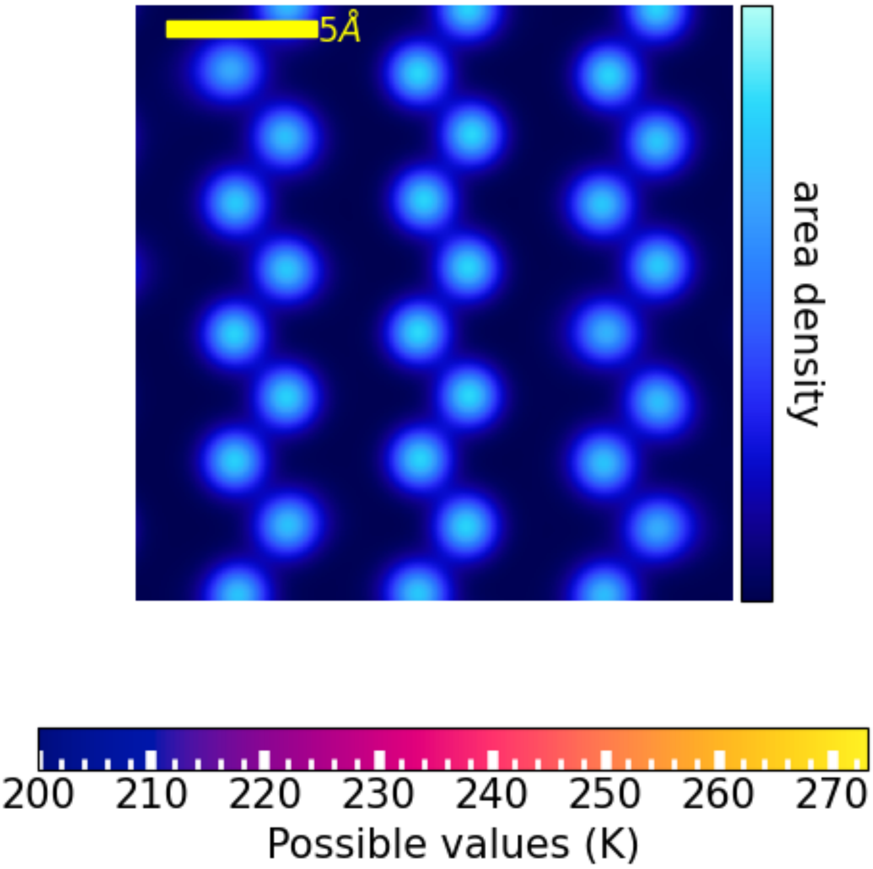
5Å
area density
200
210
220
230
240
250
260
270
Possible values (K)

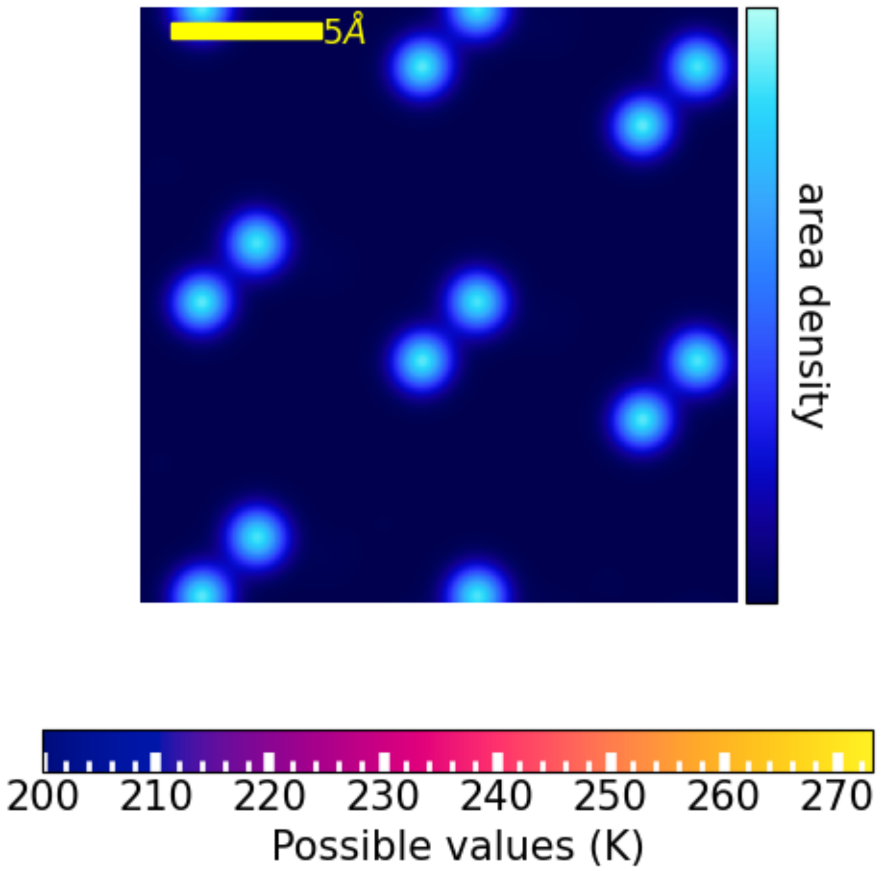

5Å
area density
200
210
220
230
240
250
260
270
Possible values (K)

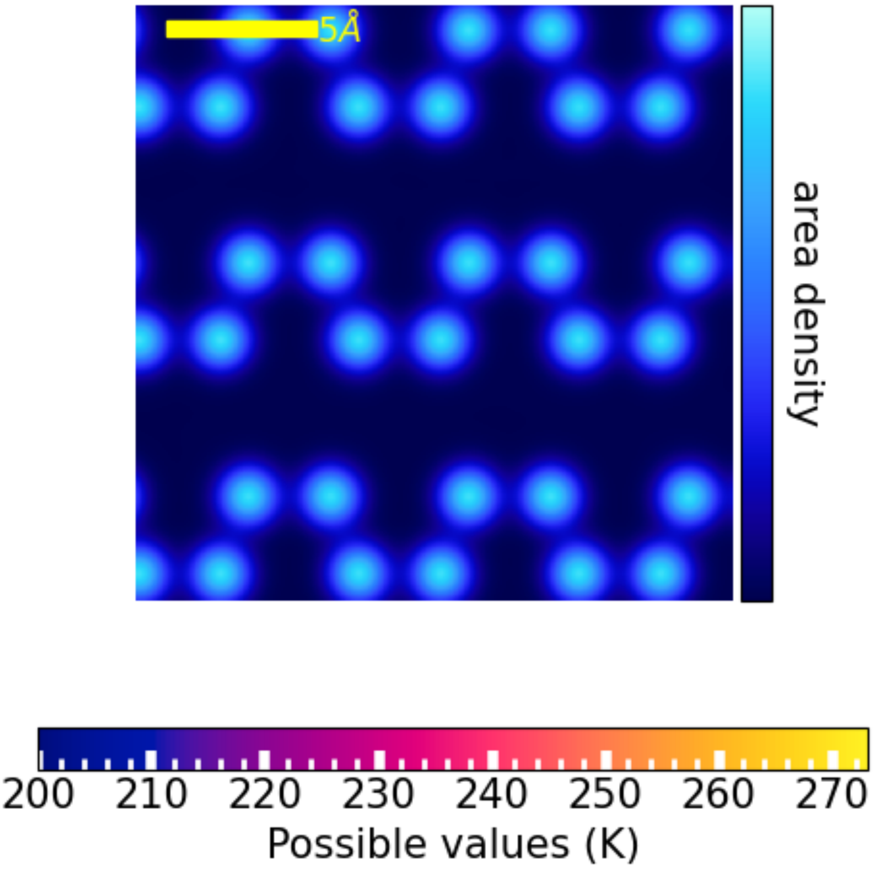
5Å
area density
200
210
220
230
240
250
260
270
Possible values (K)

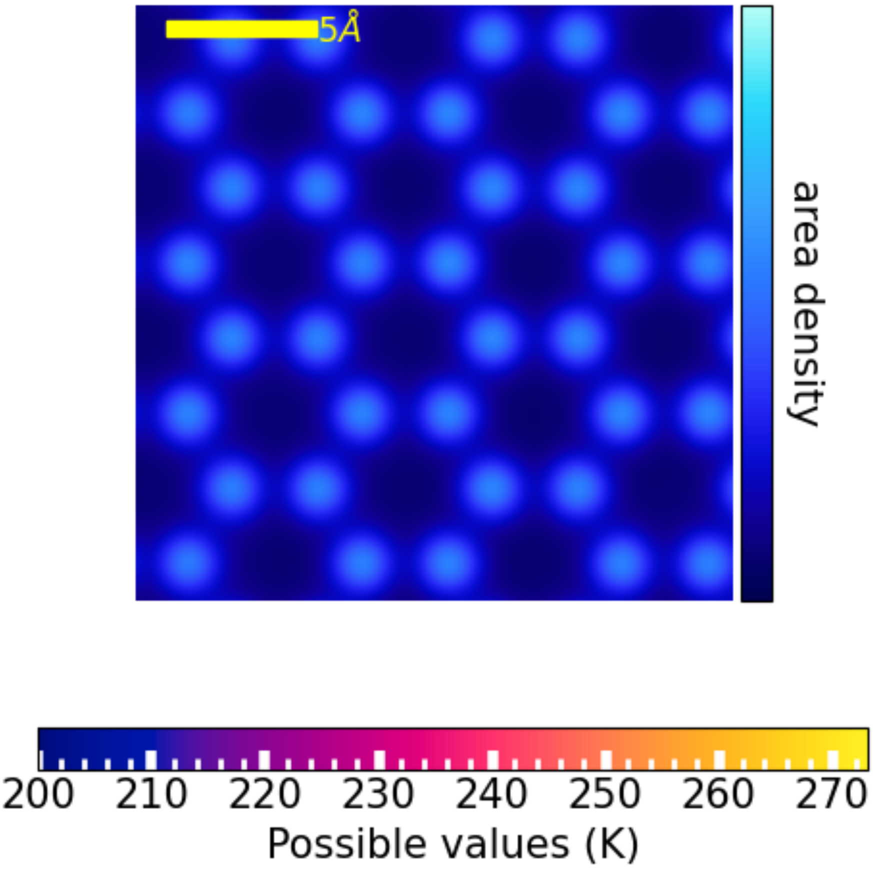

5Å
area density
200
210
220
230
240
250
260
270
Possible values (K)

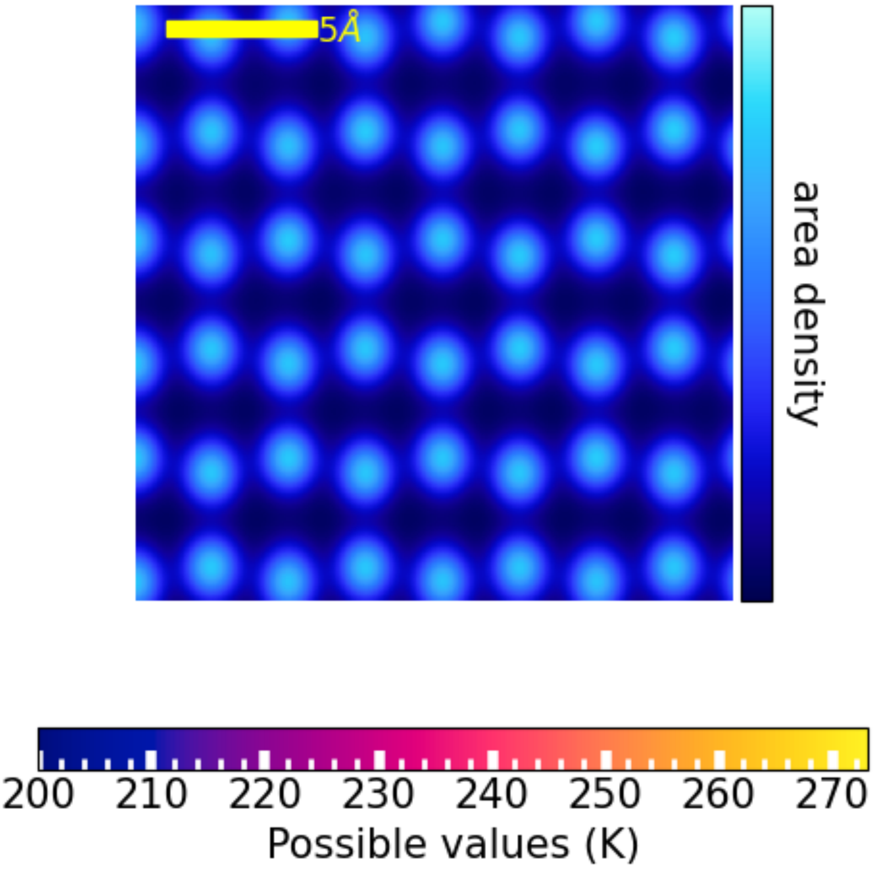
5Å
area density
200 210 220 230 240 250 260 270
Possible values (K)

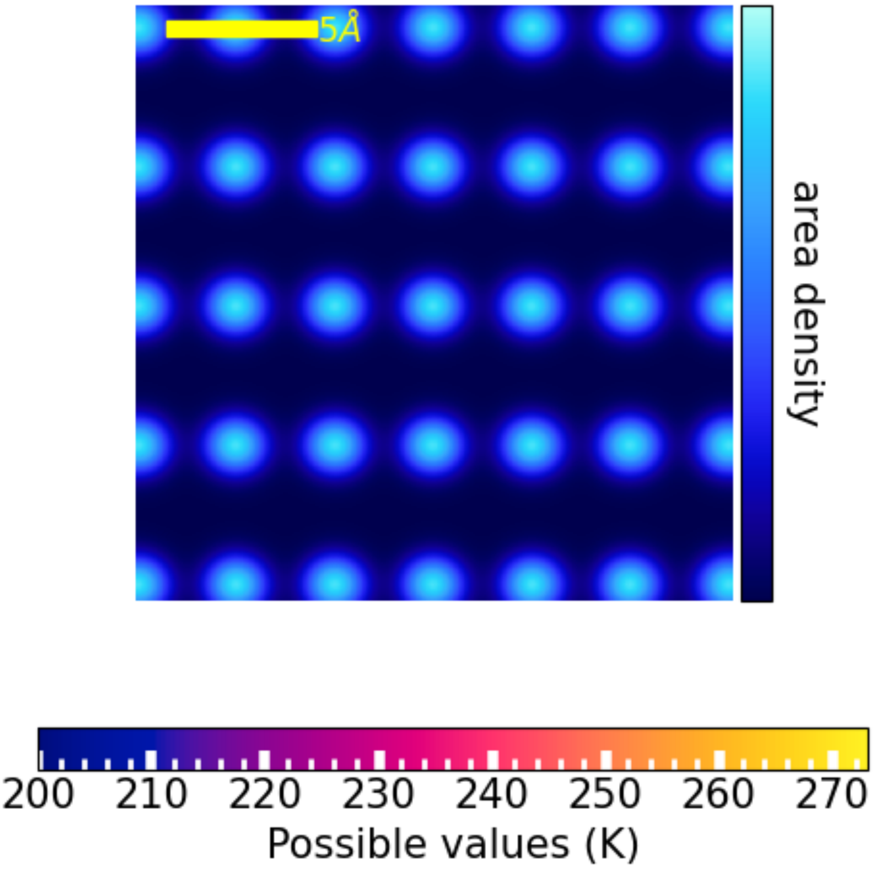

5Å
area density
200
210
220
230
240
250
260
270
Possible values (K)

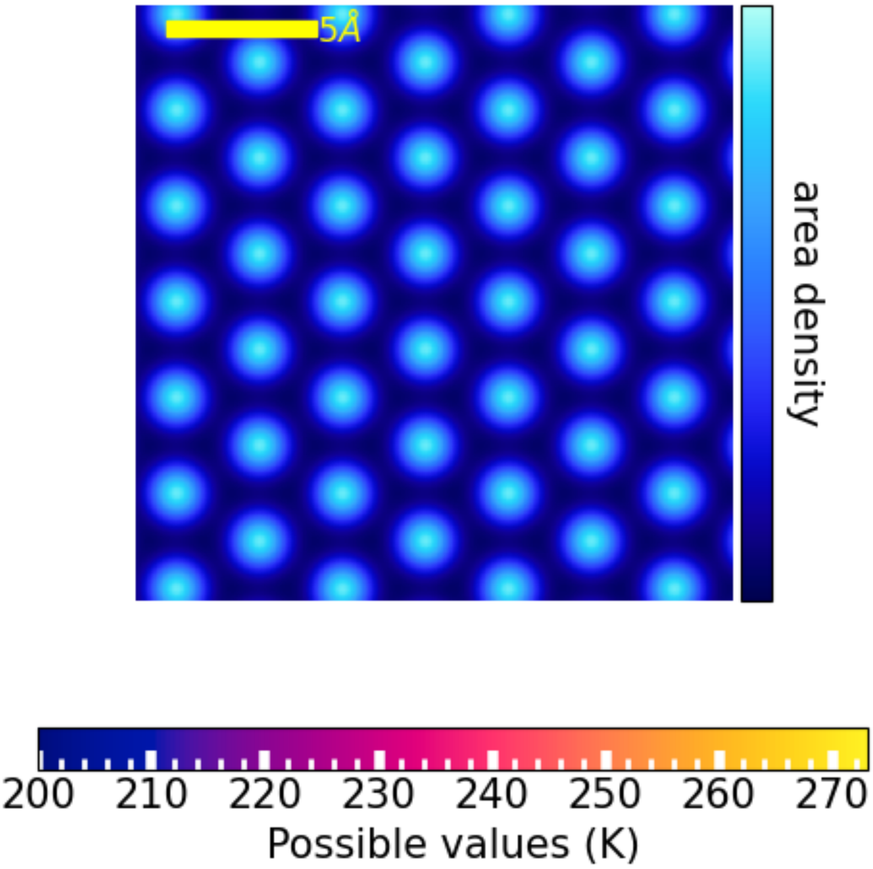
5Å
area density
200
210
220
230
240
250
260
270
Possible values (K)

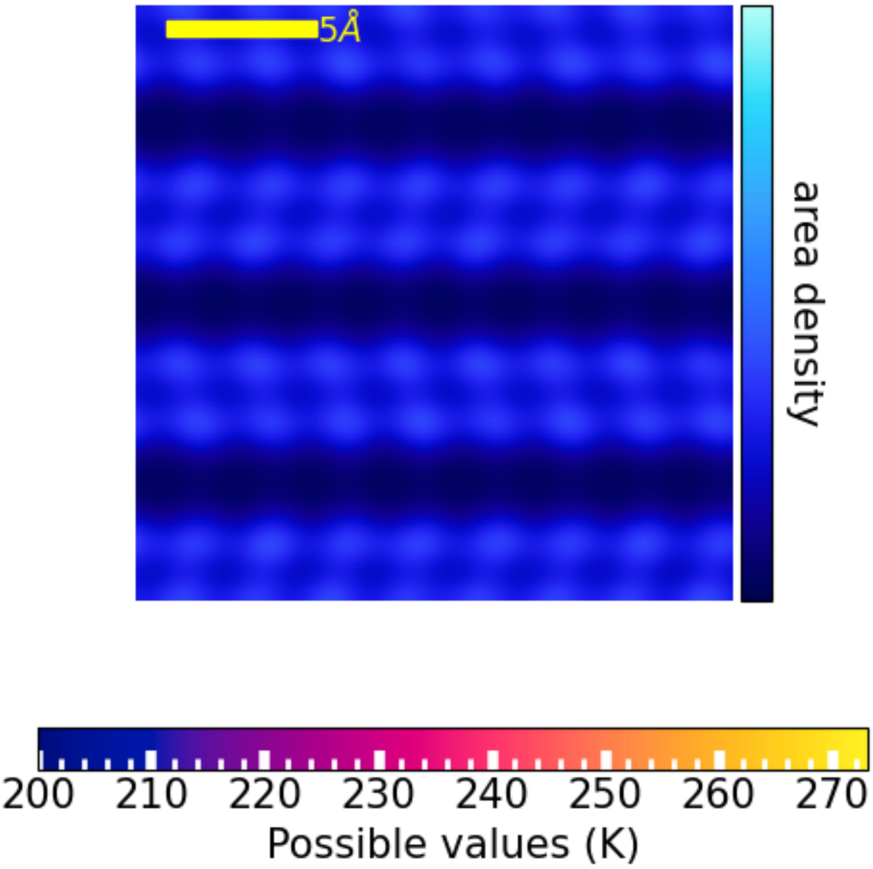

5Å
area density
200 210 220 230 240 250 260 270
Possible values (K)

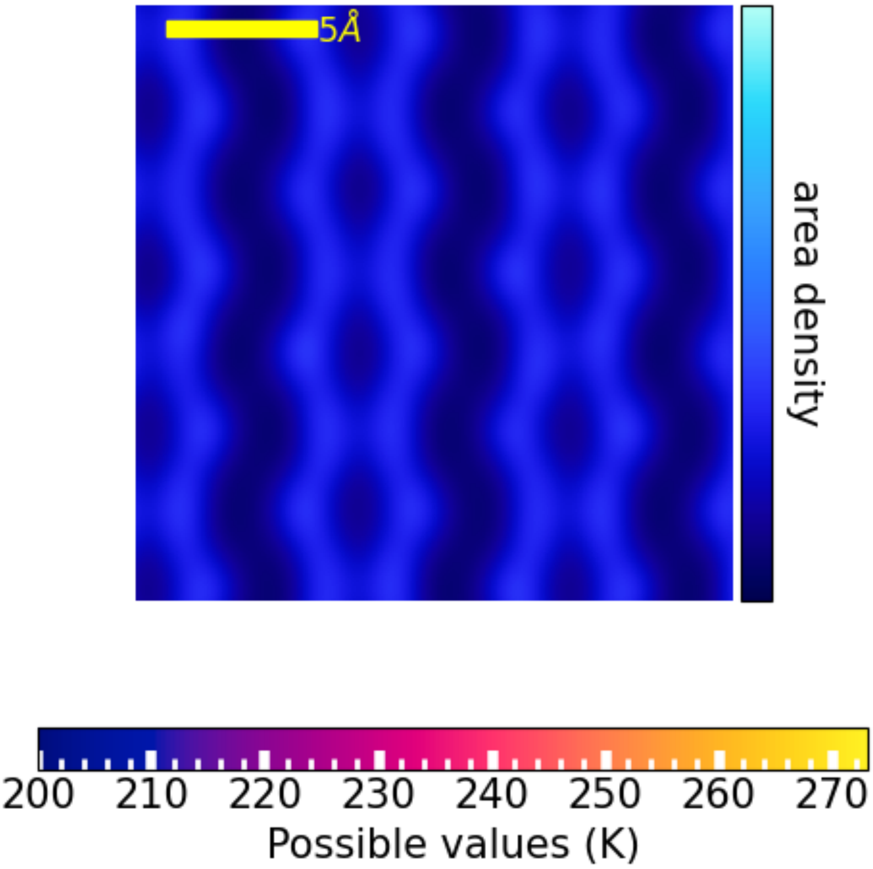
5Å
area density
200
210
220
230
240
250
260
270
Possible values (K)

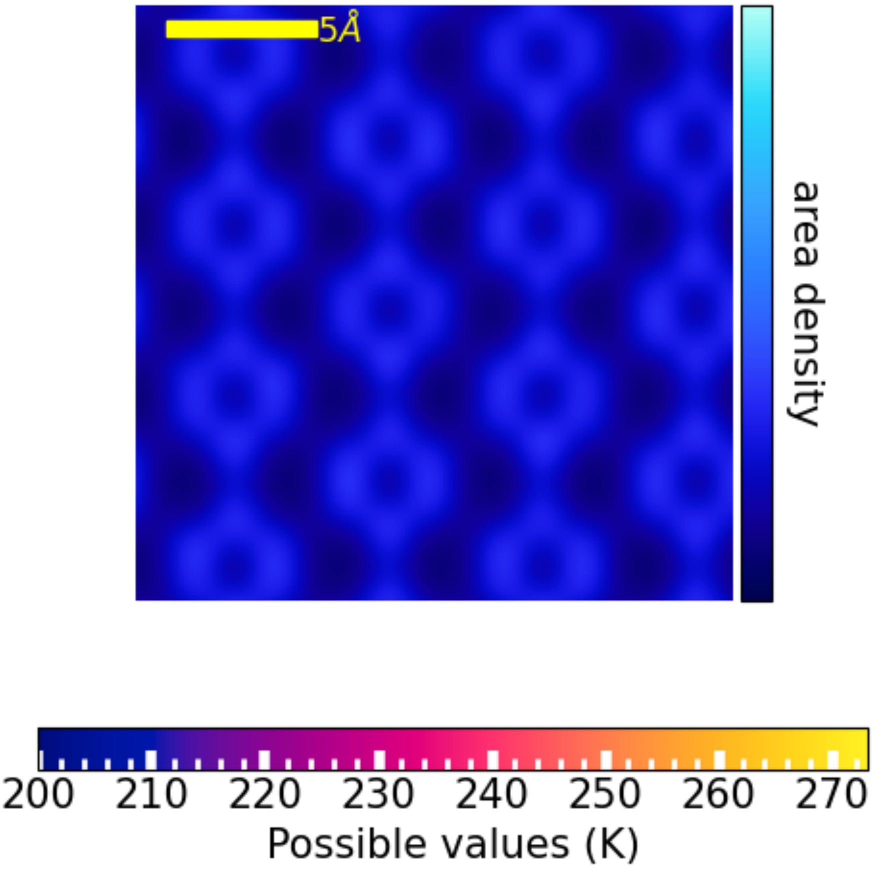
5Å
area density
200
210
220
230
240
250
260
270
Possible values (K)

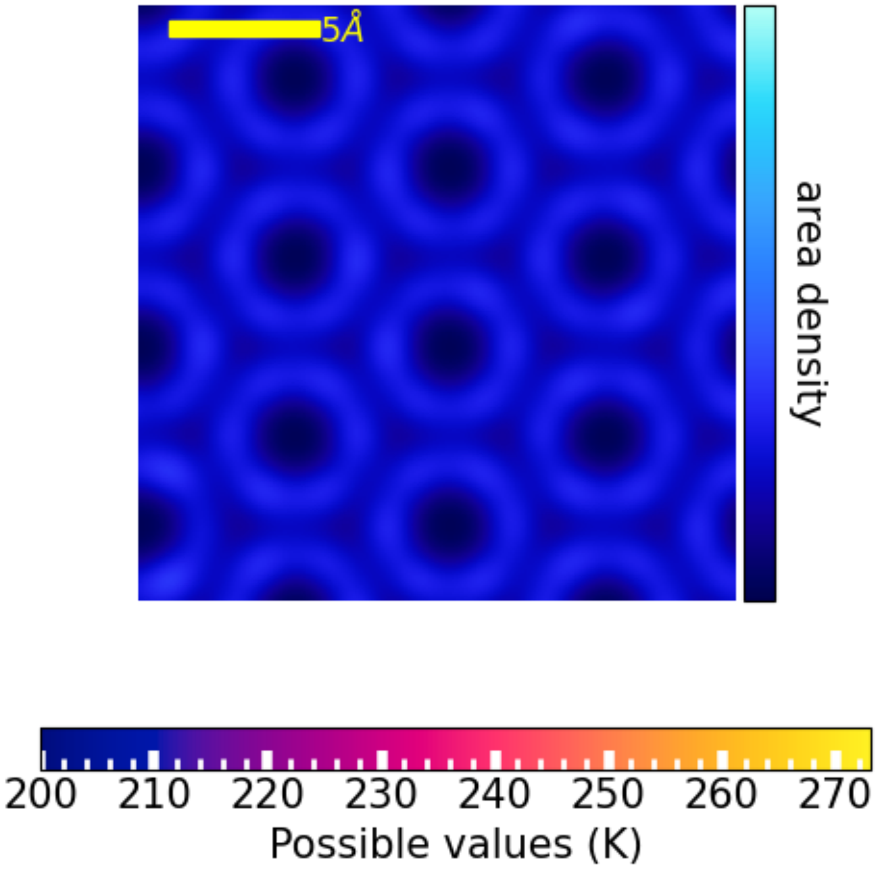
5Å
area density
200
210
220
230
240
250
260
270
Possible values (K)

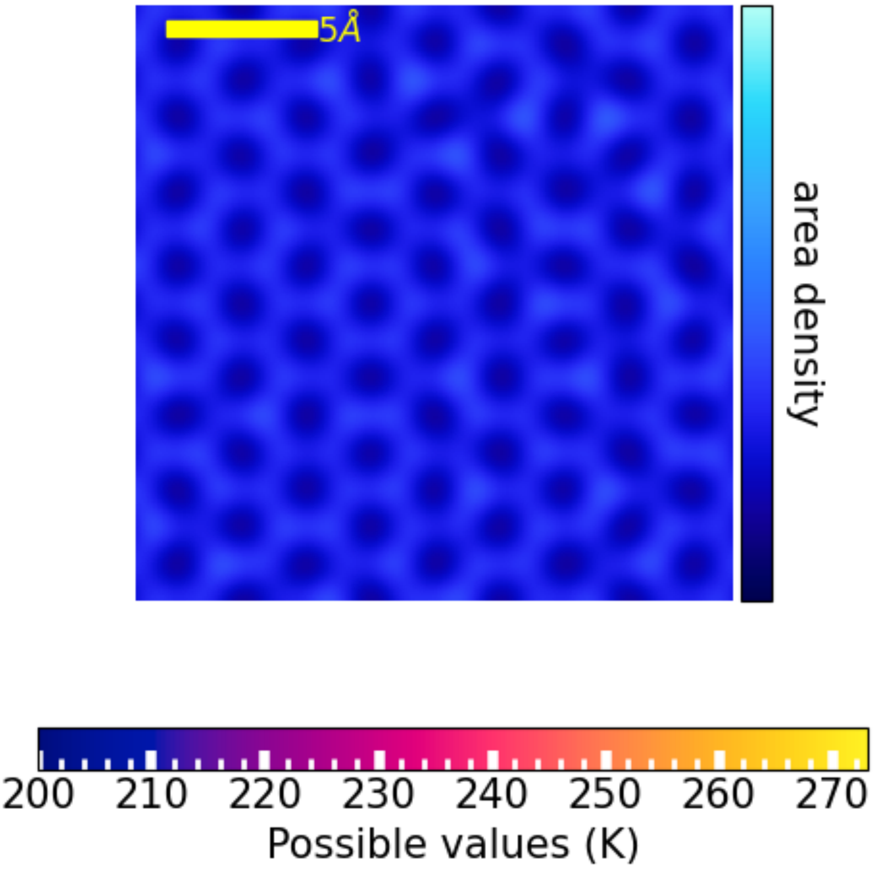
5Å
area density
200
210
220
230
240
250
260
270
Possible values (K)

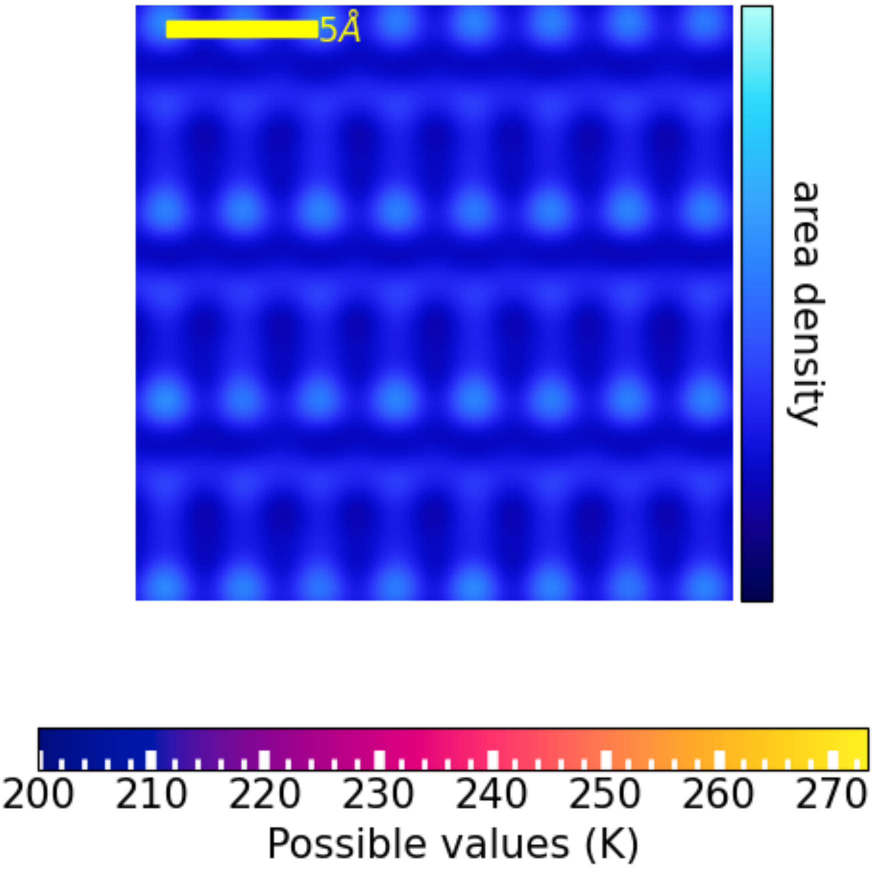
5Å
area density
200
210
220
230
240
250
260
270
Possible values (K)

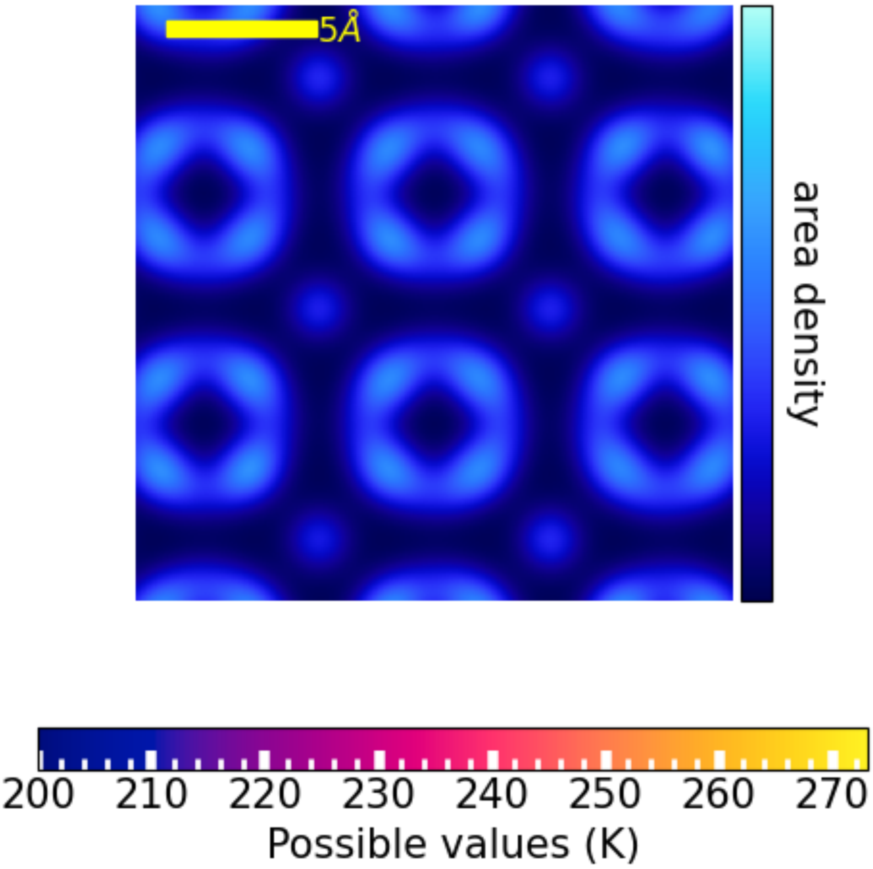

5Å
area density
200 210 220 230 240 250 260 270
Possible values (K)

Image	T
1	271
2	270
3	272
4	265
5	212
6	223
7	250
8	202
9	251
10	201
11	200
12	200
13	233
14	216
15	200
